# Genomic evidence of speciation reversal in ravens

**DOI:** 10.1038/s41467-018-03294-w

**Published:** 2018-03-02

**Authors:** Anna M. Kearns, Marco Restani, Ildiko Szabo, Audun Schrøder-Nielsen, Jin Ah Kim, Hayley M. Richardson, John M. Marzluff, Robert C. Fleischer, Arild Johnsen, Kevin E. Omland

**Affiliations:** 1 Natural History Museum, University of Oslo, P.O. Box 1172 Blindern, 0318 Oslo, Norway; 2 Department of Biological Sciences, University of Maryland, Baltimore County, 1000 Hilltop Circle, Baltimore, MD 21250 USA; 3 Center for Conservation Genomics, Smithsonian Conservation Biology Institute, National Zoological Park, Washington, 20013-7012 DC USA; 4 Department of Biological Sciences, St. Cloud State University, 720 Fourth Avenue, St. Cloud, MN 56301-4498 USA; 5 Cowan Tetrapod Collection, Beaty Biodiversity Museum, University of British Columbia, 2212 Main Mall, Vancouver, BC V6T 1Z4 Canada; 6 School of Environmental and Forest Sciences, University of Washington, Box 352100, Seattle, WA 98195 USA

## Abstract

Many species, including humans, have emerged via complex reticulate processes involving hybridisation. Under certain circumstances, hybridisation can cause distinct lineages to collapse into a single lineage with an admixed mosaic genome. Most known cases of such ‘speciation reversal’ or ‘lineage fusion’ involve recently diverged lineages and anthropogenic perturbation. Here, we show that in western North America, Common Ravens (*Corvus corax*) have admixed mosaic genomes formed by the fusion of non-sister lineages (‘California’ and ‘Holarctic’) that diverged ~1.5 million years ago. Phylogenomic analyses and concordant patterns of geographic structuring in mtDNA, genome-wide SNPs and nuclear introns demonstrate long-term admixture and random interbreeding between the non-sister lineages. In contrast, our genomic data support reproductive isolation between Common Ravens and Chihuahuan Ravens (*C*. *cryptoleucus*) despite extensive geographic overlap and a sister relationship between Chihuahuan Ravens and the California lineage. These data suggest that the Common Raven genome was formed by secondary lineage fusion and most likely represents a case of ancient speciation reversal that occurred without anthropogenic causes.

## Abstract (web summary)

Speciation reversal is known mainly from recently diverged lineages that have come into secondary contact following anthropogenic disturbance. Here, Kearns et al. use genomic and phylogenomic analyses to show that the Common Raven (*Corvus corax*) was formed by the ancient fusion of two non-sister lineages of ravens.

## Introduction

Speciation does not always follow a linear bifurcating process where new species emerge after the appearance of barriers to gene flow that drive increasing reproductive isolation^1–7^. Instead, speciation can follow a complex reticulate process in which hybridisation can create, reinforce or dissolve distinctiveness at all stages of the speciation continuum^8–12^. Most empirical examples of reticulate histories involve secondary contact after some amount of reproductive isolation has evolved. In such cases, introgressive hybridisation is often limited to small fragments of the genome, involves only a few individuals or is restricted to narrow hybrid zones^9–11^. However, if reproductive isolation fails to evolve in allopatry or erodes upon secondary contact, then hybridisation can result in the collapse of formerly distinct lineages or species into a single hybrid lineage with an admixed mosaic genome^8–10,13–17^. This reticulate pathway is most often referred to as speciation reversal or lineage fusion, and it can occur at all stages of the speciation continuum, effectively ‘reversing speciation’ or causing ‘despeciation’^8,10,13^. The underlying pathway of reticulation is identical between speciation reversal and lineage fusion; however, the term speciation reversal is most often reserved for situations where the lineages were reproductively isolated prior to lineage collapse, while lineage fusion is used to describe situations where lineage collapse involves divergent lineages that were not reproductively isolated^8,13–17^.

**Fig. 1:**
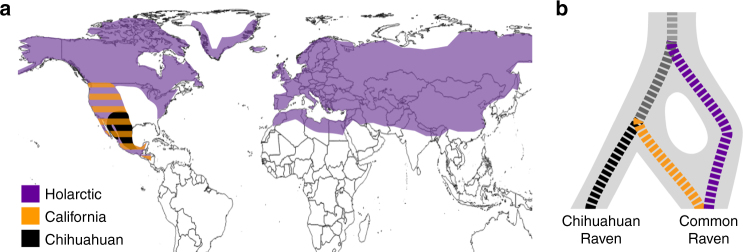
Reticulate speciation history of North American ravens. **a** Geographic range of distinct mtDNA lineages within Common and Chihuahuan Ravens based on previous mtDNA studies^18–21^ and range records^29^. **b** Hypothesis of speciation reversal where the Common Raven is formed from the fusion of non-sister California (orange) and Holarctic (purple) lineages following secondary contact, while Chihuahuan Ravens (black) remained reproductively isolated despite sympatry with the Common Raven. Dashed lines in **b** show the mtDNA gene tree topology from this and previous studies^18–21^. Solid grey background in **b** traces the changing taxonomic boundaries as the Holarctic lineage first split from the ancestor of the California and Chihuahuan lineages, and then the California and Holarctic lineages fused into a single admixed lineage

Most known cases of speciation reversal involve reproductively isolated lineages at the early stages of speciation (divergences ~1–50 kya) and many are linked to recent anthropogenic causes such as climate change and habitat loss^13–16^. Few cases of speciation reversal involving deeply diverged lineages have been identified, yet these are crucial for understanding the potential consequences of emerging cases that might be caused by anthropogenic changes in the future. Of particular concern is disentangling why some species maintain or develop reproductive isolation after secondary contact, while others experience rampant hybridisation and ‘despeciate’. Here, we use a natural experiment involving three deeply diverged lineages of ravens in North America—the ‘California’ lineage, ‘Holarctic’ lineage and Chihuahuan Ravens. Previous studies suggest a reticulate history involving two contrasting situations—(1) speciation reversal of phenotypically cryptic, but deeply divergent, California and Holarctic lineages within the Common Raven, and (2) strong reproductive isolation of phenotypically distinctive Common Ravens and Chihuahuan Ravens despite a sister relationship of the California lineage and Chihuahuan Ravens^18–21^ (Fig. 1).

Common Ravens are large non-migratory passerines that occur in most habitats across the Northern Hemisphere (Fig. 1). Previous studies identified extensive geographic overlap of two paraphyletic mtDNA lineages in the Common Raven^18–21^—the Holarctic lineage is found range-wide and the California lineage is restricted to western North America (Fig. 1). The Chihuahuan Raven is restricted to mostly arid and semi-arid grassland habitats in the southwestern United States and Mexico where it is sympatric with Common Ravens with both Holarctic and California mtDNA (Fig. 1). Despite sympatry and a sister relationship with the California lineage based on mtDNA^18–21^, Chihuahuan Ravens and Common Ravens appear to be reproductively isolated in that there are no field observations of hybridisation nor evidence of mtDNA introgression, and the two species show differences in habitat use, timing of breeding, and vocalisations in sympatry^18,19,21,22^. In contrast, tests of morphometric, behavioural and ecological trait differences between pairs of Common Ravens that had either the same mtDNA lineage or mismatched mtDNA lineages found no evidence of assortative mating, selection against hybrids or phenotypic differentiation between California and Holarctic mtDNA clades^20^. These mtDNA patterns suggest that the speciation history of Common Ravens is not strictly bifurcating, and instead could involve the fusion of distinct California and Holarctic lineages via a process of speciation reversal (Fig. 1)^18–21^.

Critically, we have not adequately tested the hypothesis of speciation reversal in Common Ravens owing to limited data from the nuclear genome in our previous studies^18–21,23–25^. The one nuclear intron examined to date showed no evidence of nuclear structuring (beta-fibrinogen intron 7^19^), whereas only one of three microsatellites examined range-wide showed allele frequency differences between California and Holarctic lineages and a high *F*_ST_ value (*F*_ST_ = 0.13, *p* < 0.0001)^18^. Thus, it remains unclear whether speciation reversal has eroded the phylogenetic signal of previously distinct California and Holarctic lineages in the nuclear genome, or if the nuclear loci examined lack  the necessary phylogenetic power, or alternatively, if mtDNA introgression involving Chihuahuan Ravens could explain mtDNA paraphyly. The latter hypothesis particularly requires testing since maternally inherited mtDNA loci are notoriously poor at reconstructing complex reticulate histories, and can lead to erroneous inferences of speciation history in such cases^11^. At a finer scale, it is also unclear whether breeding pairs with mismatched mtDNA examined by Webb et al.^20^ also have mismatched nuclear genomes. Thus, it remains to be tested whether previous inferences about random interbreeding^20^ and mitonuclear compatibility^26^ can be applied across the range of Common Ravens or if they are only applicable in the focal population examined by Webb et al.^20^ in Washington state, United States.

Here, we combine dense geographic sampling of nuclear genomes and mtDNA to test whether California and Holarctic lineages have undergone speciation reversal, and to determine whether there is genomic evidence for reproductive isolation versus random interbreeding between North American raven lineages. We show that Common Ravens in western North America have extensively admixed genomes formed from the fusion of non-sister California and Holarctic lineages into a single, randomly interbreeding, species. Two dominant phylogenies are present in their genomes—one reflecting extensive introgression and lineage fusion of California and Holarctic lineages, and the other reflecting the original bifurcating speciation history prior to lineage fusion. Genomic evidence supports current reproductive isolation between Common Ravens and Chihuahuan Ravens despite long-term sympatry and a more recent divergence of Chihuahuan Ravens and the California lineage. These lines of evidence offer strong evidence for a conclusion of speciation reversal in Common Ravens. Our study offers one of the best-characterised examples of the genomic consequences of ancient lineage fusion/speciation reversal, and thus advances our understanding of the diversity of evolutionary pathways that lineages can follow through time.

## Results

### Concordant signals of distinct lineages across the genome

**Fig. 2:**
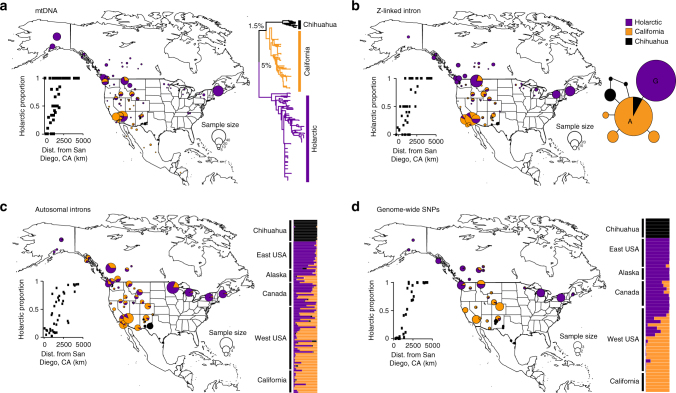
Genomic evidence of speciation reversal in Common Ravens. **a** MtDNA (*n* = 441), **b** Z intron *ACO1* (*n* = 228), **c** seven autosomal introns  (*n* = 98) and **d** genome-wide SNPs (*n* = 53; 30% missing data set: 1737 SNPs) show broadly concordant geographic structuring of California (orange), Holarctic (purple) and Chihuahuan Raven (black) lineages. Maps show the proportion of individuals in each population assigned to three distinct raven lineages with the size of each circle proportional to sample size (see inset for key). Assignment of individuals to each lineage is based on RAXML analysis shown in **a** for mtDNA, the unrooted allele network shown in **b** for the Z intron, and probability of assignment to genetic clusters corresponding to Holarctic, California and Chihuahuan in STRUCTURE analyses shown in **c** for autosomal introns and **d** for SNPs. Graphs in each show changes in the proportion of Holarctic ancestry with increasing distance from San Diego, California (calculated 'as-the-crow-flies'). Populations in Mexico and eastern United States are omitted from the graphs for better interpretability. See Supplementary Figs. 1–4 for further details of analyses

We genotyped ravens across their range in North America for mtDNA control region (Common Raven *n* = 441; Chihuahuan Raven *n* = 28), the *ACO1* nuclear intron on the Z sex chromosome (Common Raven *n* = 218; Chihuahuan Raven *n* = 20), seven autosomal nuclear introns (Common Raven *n* = 98; Chihuahuan Raven *n* = 14) and 2250 genome-wide SNPs using a double-digest restriction site-associated DNA (ddRAD) protocol (Common Raven *n* = 47; Chihuahuan Raven *n* = 6) (see Supplementary Data 1 for sample details). MtDNA, genome-wide SNPs and nuclear introns all supported the distinctiveness of three lineages of ravens in North America, which are concordant with the California, Holarctic and Chihuahuan lineages first identified by mtDNA^18–21,23–25^ (Fig. 2).

Geographic structuring within Common Ravens in all genomic data sets clearly delimits (1) a region with higher frequency of Holarctic ancestry in Canada, Alaska and the eastern United States, (2) a region with higher frequency of California ancestry in the southwestern United States and (3) a broad region of admixed California/Holarctic ancestry in the western United States. Admixed western United States populations formed a south–north clinal gradient of increasing Holarctic ancestry connecting discrete California and Holarctic clusters in all analyses (Fig. 2 and Supplementary Figs. 1–4). This gradient is a classic feature of an admixed population/lineage formed following secondary contact between previously distinct parent lineages^12^. Common Ravens from the western United States were also identified as being significantly admixed from California and Holarctic regions in a three-population test for admixture^27,28^ using the genome-wide SNP data set (f3 statistic = −0.0009 ± 0.0003, *Z* score = −2.72). No other regions/lineages were identified as admixed by three-population tests, including those for Chihuahuan Ravens and either Common Raven lineage (California vs. Holarctic, west: f3 = 0.0061 ± 0.0005, *Z* score = 11.8; Holarctic vs California, west: f3 = 0.0054 ± 0.0001, *Z* score = 43.8; Chihuahuan vs. California, Holarctic: f3 = 0.0513 ± 0.0036, *Z* score = 14.2; California vs. Chihuahuan, Holarctic: f3 = 0.0041 ± 0.0017, *Z* score = 2.43; Holarctic vs. California, Chihuahuan: f3 = 0.0069 ± 0.0024, *Z* score = 2.86).

### Details of geographic structuring

Of the 441 Common Ravens sampled from North America, 281 had Holarctic mtDNA and 159 had California mtDNA (Fig. 2 and Supplementary Fig. 1). In northwestern USA, Holarctic mtDNA had the highest frequency (*n* = 94, average frequency = 40% California, 60% Holarctic), whereas in southwestern USA and Mexico, California mtDNA had highest frequency (*n* = 121, average frequency = 61% California, 39% Holarctic). However, there was substantial variation between states and populations within each state (Fig. 2). California mtDNA was only found in two out of the 172 Common Ravens sampled from northern and eastern North America. Both of these ravens shared the same California mtDNA haplotype and came from Bowen Island near Vancouver in southwestern Canada (Fig. 2 and Supplementary Fig. 2).

Common Ravens had two major *ACO1* Z intron alleles that were divided by a single A/G SNP (Fig. 2b). Northern and eastern regions where there are high frequencies of Holarctic mtDNA were dominated by the G allele, while the A allele had higher frequencies in the southwestern United States, where the California mtDNA clade is restricted (Fig. 2b). We found 27 heterozygotes for this SNP—21 were from the western United States, five were from California and one was from Alberta, Canada. Only two individuals from northern and eastern regions were homozygous for the ‘California’ A SNP (ALB01 from Alberta, Canada and MN1559 from Minnesota, USA) (Fig. 2b).

All seven autosomal introns showed extensive sharing of alleles among the three raven lineages, however, allele frequency differences were present in several loci (Supplementary Fig. 2). STRUCTURE analyses of the autosomal introns and the genome-wide SNPs each identify two populations separating Chihuahuan Ravens and Common Ravens with little-to-no admixture as the most likely number of clusters (for details of Delta K/mean LnP(K) values see Supplementary Figs. 2 and 3). Along with the non-significant three-population tests, this is consistent with the idea that shared alleles among Chihuahuan Ravens and Common Ravens in the Z intron (Fig. 2) and all seven autosomal introns (Supplementary Fig. 2) more likely results from incomplete sorting of ancestral alleles rather than introgressive hybridisation since most shared alleles are internal in the allele networks^25^.

Under a STRUCTURE model of three populations (K3), both autosomal introns and SNPs inferred two clusters delineating pure Holarctic individuals from northern and eastern North America and pure California individuals from the state of California, and place individuals from the western United States in a gradient that connects the pure California and Holarctic clusters (Fig. 2 and Supplementary Figs. 2 and 3). Identical population structuring between California and Holarctic lineages is found by STRUCTURE and PCA when Chihuahuan Ravens are excluded, and allowing different amounts of missing data in the SNP data set (Supplementary Fig. 3). Notably, the genome-wide SNPs showed the steepest south–north transition from California to Holarctic ancestry and found little Holarctic admixture in the southwest United States compared to mtDNA, Z intron and autosomal introns (Fig. 2). However, all nuclear data sets, including the genome-wide SNPs inferred a higher proportion of California ancestry north and east into Alberta, Canada and Minnesota, USA compared to mtDNA (Fig. 2).

### Discordant ancestry assignments within individual genomes

**Fig. 3:**
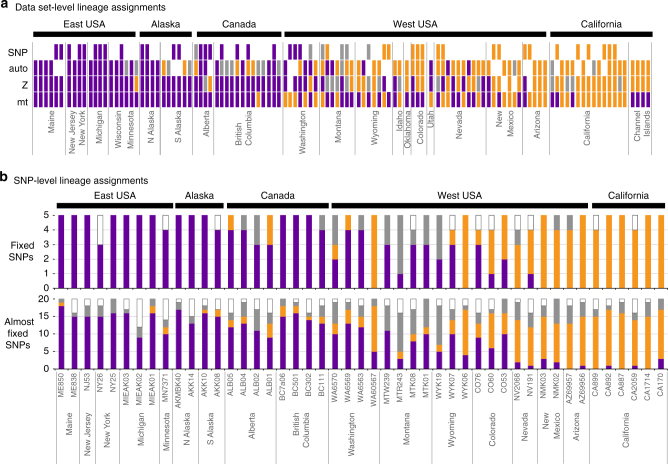
Mosaic genomes and extensive admixture within Common Ravens. **a** Genomic profiles of 116 Common Ravens from North America sampled in three or more data sets. Each box represents the assignment of an individual specimen as Holarctic (purple), California (orange) or admixed California/Holarctic (grey) based on mtDNA, Z intron, autosomal introns and SNPs (acronyms: mt, Z, auto and SNP). White indicates missing information. Specimens were designated admixed if they had probability assignments under 0.7 for both Holarctic and California clusters in STRUCTURE analyses of autosomal introns and SNPs, and were heterozygote at the A/G SNP in the Z intron. **b** California/Holarctic assignment for SNPs that was fixed (*F*_ST_ = 1; *n* = 5 SNPs) or almost fixed (*F*_ST_ > 0.58; *n* = 20) between pure individuals from the California and Holarctic lineages

Despite broadly concordant geographic signatures of lineage fusion of California and Holarctic lineages in each data set (Fig. 2), California and Holarctic ancestry based on mtDNA and nuclear data sets was uncoupled within individuals and populations across the western United States (Fig. 3a). For example, all individuals from the Channel Islands in California have pure California Z and autosomal intron profiles with Holarctic mtDNA, whereas individuals in Washington have almost no California Z alleles despite having similar Holarctic/California mtDNA proportions compared to neighbouring regions (Figs. 2 and 3a). Individuals from the western United States with California or Holarctic mtDNA were found to have almost all combinations of California or Holarctic nuclear backgrounds (Fig. 3a). Such uncoupling of California and Holarctic ancestry was evident even among SNPs that show the highest differentiation between California and Holarctic lineages (Fig. 3b). For example, the few SNPs that were fixed (*F*_ST_ = 1.0, *n* = 5 SNPs) or highly differentiated (*F*_ST_ > 0.58; *n* = 20 SNPs) between individuals with the purest Holarctic (from ME, NJ, NY populations) and purest California ancestry had mixed lineage assignments within individuals from admixed regions in the western United States and Canada—i.e., individuals had a mixture of homozygous California SNPs, homozygous Holarctic SNPs and heterozygous California/Holarctic SNPs (Fig. 3b).

### Genomic landscape of lineage fusion

**Fig. 4:**
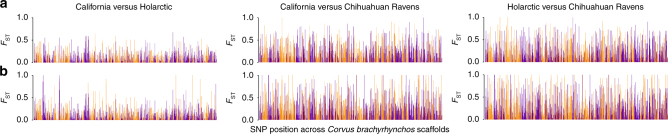
Genome-wide patterns of divergence between North American raven lineages. Manhattan plots show mean pairwise *F*_ST_ for each SNP estimated between each lineage using data sets that either **a** excluded (*n* = 2689 SNPs) or **b** included (*n* = 2969 SNPs) SNPs that violated Hardy–Weinberg equilibrium (HWE). Scaffolds are ordered numerically across the *Corvus brachyrhynchos* genome and neighbouring scaffolds are identified by alternating purple and orange colouration

Estimates of mean pairwise *F*_ST_/*ϕ*_ST_ and genetic diversity differed between the three raven lineages across the data sets (Supplementary Tables 1 and 2). Divergences were similarly high between Chihuahuan and Common Raven lineages (mtDNA = 0.54–0.75, *Z* = 0.70–0.94) compared to between California and Holarctic lineages (mtDNA = 0.59, *Z* = 0.93) for mtDNA and Z intron, but were more than twice as high between Chihuahuan and Common Raven lineages (autosomal = 0.31–0.41, SNPs = 0.17–0.21) compared to between California and Holarctic lineages (autosomal = 0.15, SNPs = 0.04) for autosomal introns and SNPs. The genomic landscape of divergence was similar between Chihuahuan Ravens compared to either Holarctic or California lineages with generally high *F*_ST_ across the genome and shared peaks of divergences (Fig. 4). *F*_ST_ was substantially lower across the genome between Holarctic and California lineages. Some peaks of divergence shared between the Common Raven lineages versus Chihuahuan Raven were also present between California and Holarctic lineages. Critically, we found that SNPs with the highest *F*_ST_ violated HWE and were not present in the stringently filtered SNP data set (Fig. 4). This had the most impact on the pairwise comparison of California and Holarctic lineages, where all SNPs with *F*_ST_ > 0.68 violated HWE (Fig. 4a).

### Discordant phylogenetic signals across the genome

**Fig. 5:**
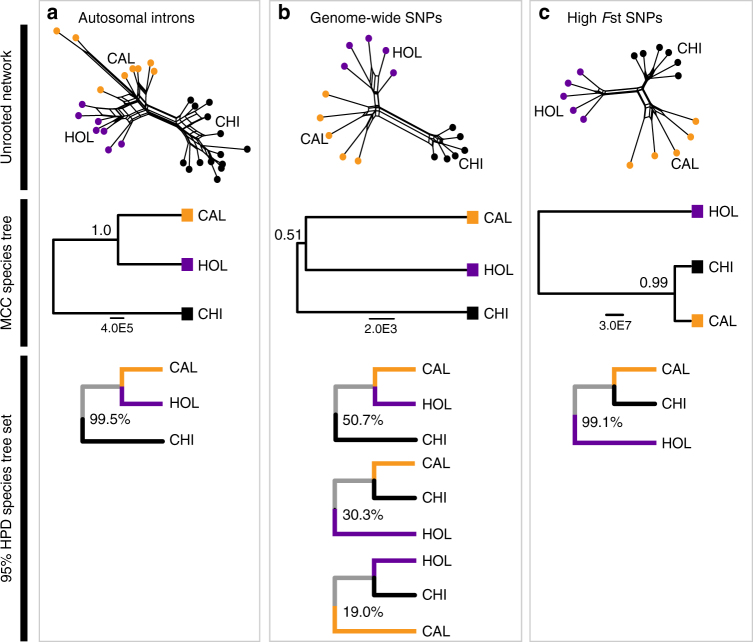
Discordant phylogenetic signals in the nuclear genome. Unrooted Neighbour-Net trees and species trees inferred for the three lineages of North American ravens—Chihuahuan Ravens (CHI, black), pure Holarctic (HOL, purple) and California (CAL, orange)—estimated from three nuclear data sets: **a** autosomal introns, **b** all genome-wide SNPs, **c** high *F*_ST_ SNPs between California and Holarctic lineages (*n* = 131 SNPs with *F*_ST_ > 0.2). For species trees, we show the maximum clade credibility (MCC) species tree inferred from all post-stationarity trees, and the percentage of trees matching each topology contained within the 95% HPD set of trees in each data set

MtDNA in this and previous studies^18–21,24^ strongly support a sister relationship between the California lineage and Chihuahuan Ravens (Fig. 2a and Supplementary Fig. 1). The *ACO1* Z intron also shows a close relationship between the California lineage and Chihuahuan Ravens (Fig. 2b). In contrast, phylogenetic analyses of genome-wide SNPs and autosomal introns using both unrooted Neighbour-Net and species tree approaches find a close relationship between California and Holarctic lineages, and differentiate Common and Chihuahuan Ravens as distinct lineages—i.e., they support the reciprocal monophyly of Common Ravens (Fig. 5a, b). California and Holarctic lineages emerge as slightly distinct lineages in Neighbour-Net trees of genome-wide SNPs and autosomal introns that included only pure individuals (Holarctic: all individuals from ME, NJ, NY; California: all individuals from CA except slightly admixed CAIS1, CAIS2, CAIS3, CAIS4, CA895, CA881 and NCA56) (Fig. 5), however, this distinctiveness erodes when admixed individuals from the western United States and Canada are included (Supplementary Figs. 2c and 4).

Species trees estimated from autosomal introns strongly supported a sister relationship between California and Holarctic lineages (Fig. 5a; 99.5% of topologies within the 95% HPD set of trees; posterior probability support in the maximum clade credibility (MCC) species tree = 1.0), however, the genome-wide SNP data set had poor support for this relationship (Fig. 5b; posterior probability support in the MCC tree = 0.51). Instead, three dominant species tree topologies were present in the genome-wide SNP data set based on the 95% HPD set of trees—50.7% supported California and Holarctic as sister taxa, 30.3% supported California and Chihuahuan Ravens as sister taxa and 19.0% supported Holarctic and Chihuahuan Ravens as sister taxa (Fig. 5b). Species tree and Neighbour-Net analyses estimated from SNPs that showed the least admixture between California and Holarctic lineages (131 SNPs with *F*_ST_ > 0.2) strongly supported a sister relationship between California and Chihuahuan Ravens (Fig. 5c; 99.1% of topologies in the 95% HPD set of trees; posterior probability support in the MCC tree = 0.99)—this topology is concordant with the mtDNA phylogeny (Fig. 2a).

### Suitability of Pleistocene climates

**Fig. 6:**
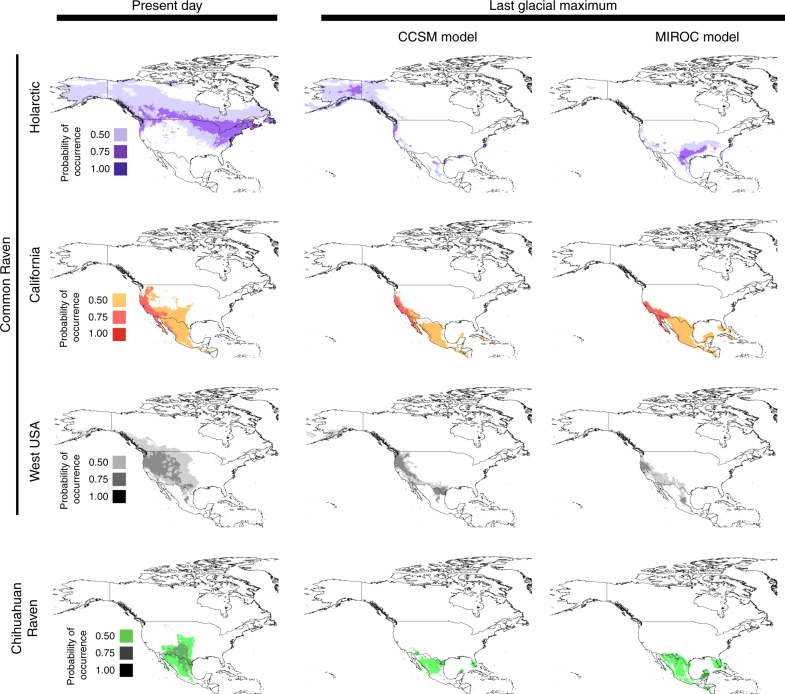
Suitable climatic niches for North American raven lineages under present-day and Pleistocene LGM (18–21 kya) climatic conditions. ENMs predict overlap in suitable climatic niches in western United States, Mexico and Central America for Holarctic, California and Chihuahuan Raven lineages during the LGM under both MIROC and CCSM paleo-climate models. Colours correspond to the predicted relative climatic suitability for each lineage in North America (see inset for key to probability of occurrence). Regions with probability of occurrence above 0.4 are considered typical of the abiotic niche of the species. Test AUC scores for each model followed by the maximum possible AUC score in brackets and the standard deviations are as follows: California: 0.77 (0.78) ± 0.003; Holarctic: 0.64 (0.64) ± 0.003; admixed west USA: 0.71 (0.72) ± 0.003; Chihuahuan Ravens: 0.88 (0.88) ± 0.003. See Supplementary Fig. 5 for ENMs based on different regional subsets and for collection localities used in ENMs for each lineage

Present-day ecological niche models (ENMs) predicted a slightly more extensive range for Common Ravens than predicted based on occurrence records and field observations (Fig. 1; see also ref. ^29^). This could signify issues with model fit associated with differentiating absence vs. presence records and the broad range of climatic conditions covered by the localities used, however, it could also indicate that the range of Common Ravens is limited by other variables that were not included in our models (e.g., such as tree cover and human extirpation). Notably, overlaying separate ENM predictions for Holarctic, western United States and California regions (Fig. 6) offer a better fit to the species range than including all localities in a single model (Supplementary Fig. 5). Nonetheless, all present-day ENMs for each lineage/region had AUC scores above the 0.5 threshold indicating that they performed better than random chance (AUC = 0.57–0.95) (Fig. 6 and Supplementary Fig. 5). The lowest values were for ENMs based on all Common Raven localities (Supplementary Fig. 5: AUC = 0.57) and those including widespread Holarctic localities (i.e., Holarctic region alone: AUC = 0.64, Fig. 6; Holarctic and western USA combined: AUC = 0.59, Supplementary Fig. 5). This fits with known issues of applying AUC scores to species with widespread distributions or extensive presence records within the region of interest^30–33^.

Paleo-ENM predictions from two different climatic models (CCSM and MIROC) for the last glacial maximum (LGM, 18–21 ka) differed substantially for the Holarctic lineage (Fig. 6 and Supplementary Fig. 5). CCSM predicted a broader range of suitable climates including extensive suitable regions in northwestern Canada and Alaska for all Common Ravens, and for the Holarctic and western United States subsets of the data set. In contrast, MIROC predicted only a small region of suitable habitat for Common Ravens in northern Alaska (Fig. 6 and Supplementary Fig. 5). Nevertheless, CCSM and MIROC models each predict a contraction of suitable habitats for Chihuahuan Ravens and Common Ravens during Pleistocene glacial maxima. Despite this predicted range contraction, extensive suitable habitats are still predicted for both Chihuahuan and Common Ravens in the southwestern United States and Mexico during the LGM (Fig. 6 and Supplementary Fig. 5).

### Timing of lineage fusion

MtDNA divergences among all North American raven lineages are estimated to have occurred within the past 2 million years (California—Chihuahuan Raven divergence: 0.59–1.51 mya; Holarctic—California + Chihuahuan divergence: 0.87–2.05 mya; Supplementary Fig. 1). Further, both Holarctic and California mtDNA lineages exhibit patterns of phylogeographic structuring that support the long-term presence of both lineages in western North America—both lineages have multiple haplo-groups with near or complete geographic restriction in the western United States, including two divergent Holarctic mtDNA haplotypes that are only found in California and southern Nevada (haplotypes B & J; Supplementary Fig. 1c). Paleo-ENMs predict extensive suitable habitats for all three raven lineages in the western United States during the LGM, which supports the hypothesis that all three lineages could have persisted in climatic refugia in the western United States during the Pleistocene, with Common Ravens most recently re-colonising eastern and northern North America from refugia following the amelioration of glacial conditions and retreat of ice-sheets in these regions after the LGM (Fig. 6 and Supplementary Fig. 5). Signatures of recent range-wide expansions present in the phylogeographic structuring of both California and Holarctic mtDNA lineages appears to support this—the most extensive of which involves haplo-group A in the Holarctic clade, which is characterised by a classic star-shaped polytomy and is present across all of North America including the most southern locality of the Holarctic clade at the United States–Mexico border (Supplementary Fig. 1).

## Discussion

Genomic patterns within Common Ravens in North America are consistent with a reticulate history involving the fusion of two deeply divergent non-sister lineages (Fig. 1). In Common Ravens, lineage fusion appears to have resulted in the complete or near-complete replacement of pure California genomes with substantially admixed genomes representing a mosaic of California/Holarctic ancestry (Fig. 2). Many parts of the western United States and California still have high proportions of California ancestry despite Holarctic alleles being present throughout these regions. In contrast, California alleles have not penetrated deep into the northern and eastern regions dominated by the Holarctic lineage (Figs. 2 and 3).

Patterns of phylogenetic discordance among data sets (Figs. 2 and 5) appear fully concordant with a hypothesis of continued reproductive isolation between Common Ravens and Chihuahuan Ravens despite random interbreeding and lineage fusion of non-sister California and Holarctic lineages across western Northern America (Fig. 1b). The sister relationship between California and Chihuahuan Ravens that is supported by mtDNA and a small proportion of the nuclear genome (including SNPs putatively resistant to admixture between California and Holarctic lineages; Fig. 5b, c) likely represents the original bifurcating speciation history of North American ravens that has since been eroded in the majority of the nuclear genome by substantial admixture during the fusion of California and Holarctic lineages. Thus, the phylogenetic signal shown in the autosomal and genome-wide SNP data sets, which supports a closer relationship between California and Holarctic lineages to the exclusion of Chihuahuan Ravens (Fig. 5a, b), likely reflects the more recent reticulate history of North American ravens resulting from long-term admixture and fusion of California and Holarctic lineages while Chihuahuan Ravens remained reproductively isolated. ENMs predict widespread geographic overlap between Common Raven populations dominated by California ancestry and Chihuahuan Ravens at the LGM (Fig. 6), suggesting that long-term sympatry, possibly since their initial divergence (0.59–1.51 mya; Supplementary Fig. 1), could have acted to reinforce their reproductive isolation. Notably, the presence of a sister relationship between California and Chihuahuan Ravens in parts of the nuclear genome strongly argues against the alternative hypothesis that mitonuclear discordance results from the replacement of parental Chihuahuan mtDNA following ancient mtDNA capture with the California lineage.

Our much denser geographic sampling of mtDNA compared to previous studies^18–21,23–25^ conclusively delineates the Vancouver region of southwestern Canada as the northern extent of the California mtDNA clade, whereas California alleles in intron and SNP data sets penetrated at low frequency further north into southern Canada (and southern Alaska for the autosomal introns) and east into Minnesota, USA (Fig. 2 and Supplementary Figs. 1–3). Such differences in rates of introgression between mtDNA and nuclear markers are expected owing to differences in their effective population size, mutation rates, selection strengths, maternal versus biparental inheritance and differences between the sexes in dispersal distance and likelihood of hybridisation^11^. In Common Ravens, more extensive northward introgression of nuclear alleles compared to mtDNA could indicate higher rates of male dispersal and/or gene flow, or selection against California mtDNA further north. Holarctic mtDNA was found as far south as the United States–Mexico border (Fig. 2a and Supplementary Fig. 1). Full characterisation of the southern extent of the Holarctic lineage awaits dense geographic sampling of mtDNA and nuclear genomes across Mexico and Central America—a region where few modern specimens have been collected. Given the clinal decrease in the frequency of Holarctic ancestry towards the southwestern United States and Mexico (Fig. 2), it is possible that pure California ancestry might still persist in southern Mexico and Central America.

The exact timing of lineage fusion remains uncertain, however, most evidence supports ancient secondary contact and long-term random interbreeding between Holarctic and California lineages. Divergence estimates from mtDNA suggest that initial secondary contact could have occurred at least as early as the mid-Pleistocene (140–440 kya) when Holarctic ravens from North America and Eurasia diverged with little-to-no subsequent gene flow^25^. If secondary contact was recent and/or if there was strong selection against hybrids, as is the case for many species that have been hybridising for long periods of time, then a narrow hybrid zone is expected^9,12^. However, California and Holarctic lineages are admixed across >1500 km of the western United States, and few, if any, regions of pure California ancestry remain. Uncoupling of mtDNA and nuclear genomes throughout the western United States (Fig. 3) also fits with a hypothesis of ancient secondary contact of California and Holarctic lineages. Such uncoupling is indicative of mosaic genomes that originate after many generations of random interbreeding without strong selection against hybrids^10^, and agrees with previous field research from populations in Washington State, USA (near the northern extent of the mtDNA zone of sympatry) that found random interbreeding between Holarctic and California mtDNA lineages and a lack of selection against hybrids^20^.

Our data appear most consistent with California and Holarctic lineages having experienced multiple secondary contact events as they tracked oscillations in Pleistocene climates, rather than a single ancient secondary contact event that initiated lineage fusion^12^. Most notably, we find both recent and ancient temporal signatures in mtDNA (Supplementary Fig. 1), geographic and genomic heterogeneity in California/Holarctic admixture across the western United States (Fig. 2), and ENM predictions that suggest extensive suitable habitats in the western United States that could have supported large admixed populations throughout the Pleistocene (Fig. 6). More recent post-LGM secondary contact and introgression of Holarctic alleles from purer northern populations with already admixed western United States populations could explain the maintenance of clinal variation in California/Holarctic admixture in the western United States despite initial secondary contact being ancient (Fig. 2). However, such clinal variation could also be maintained despite substantial admixture and ancient initial secondary contact if selection linked to mitonuclear incompatibilities and/or climatic gradients favoured California-dominated genomes in the south and Holarctic-dominated genomes in the north^26^. Complex temporal and geographic signatures of Pleistocene secondary contact in other fauna in western North America^34,35^ also implicates a role for the diverse geographic features of the western United States in further enhancing the complexity of temporal and geographic signatures of lineage fusion in Common Ravens by providing multiple Pleistocene micro-refugia, diverse barriers to dispersal (e.g., Cascade-Sierra Nevada mountains), heterogeneous vegetation types (e.g., temperate forests to deserts) and climatic gradients.

Genomic patterns of lineage fusion in Common Ravens fit with expectations of speciation reversal, wherein there should be evidence of extensive genetic swamping, replacement of one or both parent lineages, random interbreeding, and uncoupled hybrid mosaic genomes formed from two divergent parent lineages^8^. Each case of speciation reversal documented to date has different strengths of evidence for these different expectations. For example, most empirical examples of speciation reversal (e.g., sticklebacks^15^, whitefish^14^ and finches^16^) have very narrow geographic scales and thus, in these situations, speciation reversal can result in the complete replacement of both parent lineages with hybrid genomes over a short time frame. This contrasts with the raven situation, where substantial time and gene flow would be required for mosaic genomes comprised of the collapsing California and Holarctic lineages to penetrate across the entire range of Common Ravens (Fig. 1). For ravens, we also do not have direct evidence of the amount of intrinsic reproductive isolation between California and Holarctic lineages that was present prior to lineage fusion. Thus, it is not clear-cut whether we should call the situation in ravens ‘speciation reversal’ or view it as a case of ‘ancient lineage fusion’. This contrasts with most other examples of speciation reversal, where there is strong evidence for the strength and nature of reproductive isolation prior to speciation reversal despite a very shallow divergence between lineages (e.g., sticklebacks^15^). Two lines of evidence suggest that California and Holarctic lineages could have been reproductively isolated prior to secondary contact and lineage fusion. First, the timing of divergence of the Holarctic lineage and the ancestor of the California and Chihuahuan lineages between 0.9 and 2 mya (Supplementary Fig. 1) is approaching the limit where most bird taxa (especially those in the northern hemisphere) have evolved reproductive isolation (~2 mya)^36,37^. Second, life history traits^22^, as well as mtDNA, intron, SNP and ENM analyses all support reproductive isolation between Chihuahuan Ravens and Common Ravens despite the more recent divergence of the Chihuahuan Raven and the California lineage 0.6–1.5 mya (Supplementary Fig. 1). This shows that ravens can develop reproductive isolation and maintain strong species boundaries after a more recent divergence than that between California and Holarctic lineages. We argue that our findings represent the strongest support possible for the conclusion of speciation reversal given the inability to measure ancient prefusion reproductive isolation.

Complex reticulate histories are emerging all across the tree of life—including humans^1^, mosquitoes^2^, butterflies^3^, sunflowers^4^, oaks^5^, bears^6^, wolves^7^, Darwin’s finches^16^ and now ravens. These diverse examples illustrate the prevalence of reticulation as an important evolutionary process. Our work on ravens shows that while the genomic consequences of speciation reversal are discernable in the genome even after long periods of random interbreeding, detecting, characterising and unravelling such complex reticulate histories poses considerable challenges even when sophisticated genomic techniques and substantial geographic sampling are available. Relatively few examples of speciation reversal have been documented, however, this process is expected to become increasingly common as anthropogenic actions cause more species without reproductive isolation into rapid secondary contact^13–15^. Critically, although speciation reversal causes a net loss in biodiversity, the collapsing species are not remerging into their original ancestral state, but rather a new lineage is produced with a unique mosaic genome formed from its parent lineages. In this changing world, speciation reversal could create novel, but advantageous, combinations of alleles that allow mosaic hybrid species to occupy habitats and fill niches their parent species could not (e.g., coyote-wolf hybrids^7^). The challenge now is for taxonomy and conservation legislation to reconsider outdated views of evolution as a strictly bifurcating process and species as ‘end-points’ of evolution in order to better describe earth’s biodiversity and offer protection to naturally formed lineages with mosaic hybrid genomes^38^.

## Methods

### Sampling approach

Our sampling focused on increasing geographic representation of Common Ravens along the northern, southern and western borders of the western United States, where previous studies had insufficient sampling to identify the geographic extent of overlap between two divergent mtDNA clades within the Common Raven—Holarctic (HOL) and California (CAL)^18–21,23–25^ (Fig. 1). We particularly focused on obtaining the first sequences from Canada including geographically widespread sampling of provinces near the putative northern extent of the California mtDNA clade (British Columbia: *n* = 44, Alberta: *n* = 13, Saskatchewan: *n* = 5). We also provide the most geographically widespread sampling of Mexico (*n* = 14), populations east of the Sierra-Cascade mountain range in the western United States (Montana: *n* = 33, Wyoming: *n* = 22, Colorado: *n* = 4, New Mexico: *n* = 28, Utah: *n* = 8) and the eastern United States (*n* = 57). The majority of specimens used in this study were frozen liver or blood tissues obtained as loans from museums and institutions (see Supplementary Data 1). Little-to-no fresh tissue or blood samples were available for ravens in Mexico, Arizona, Utah, Colorado and Oregon, therefore we sampled small pieces of tissue from the toe-pads of museum study specimens to obtain sufficient sampling from these regions. To improve sampling coverage of Common Ravens, we collected blood samples from live birds in Montana, USA, which were captured using rocket nets and then released (Montana State permit: 2014–024; IACUC: KO 010671316; Federal permit: BB22513), and tissue samples from salvaged carcasses from British Columbia, Canada (BC) (permits: BC FLINRO Wildlife Act Permit: VI12-72390; Scientific-Salvage: BS-SA-0022-13, BS-SA-0022-14). Carcasses were salvaged from distinct ecological regions in British Columbia during a 4500-km collecting trip conducted at the peak of the raven fledgling period (last week in June–first week in July), and through donations from the BC Ministry of Forest, Lands and Natural Resources Operations, universities and wildlife rehabilitation organisations. Ethical approval: All applicable international, national and/or institutional guidelines for the care and use of animals were followed.

We genotyped mtDNA in a total of 441 Common Ravens and 28 Chihuahuan Ravens from North America using mtDNA control region sequences either produced for this study (Common Raven *n* = 218; Chihuahuan Raven *n* = 6) or published in previous studies by our group (Common Raven *n* = 238; Chihuahuan Raven *n* = 22)^18–21,23–25^. We also included previously published mtDNA sequences from Common Ravens from the Old World (*n* = 35) and Greenland (*n* = 14) for comparison. We sequenced loci from across the nuclear genome for a subset of specimens that were genotyped for mtDNA—Z sex chromosome intron (Common Raven *n* = 218; Chihuahuan Raven *n* = 20), seven autosomal nuclear introns (Common Raven *n* = 98; Chihuahuan Raven *n* = 14), and thousands of SNPs captured using a double-digest restriction site-associated DNA (ddRAD) protocol (Common Raven *n* = 47; Chihuahuan Raven *n* = 6) (see Supplementary Data 1 for sampling details). All three nuclear data sets have broad coverage of Common Ravens from the western United States (Z intron: *n* = 89; autosomal intron: *n* = 41; ddRADs: *n* = 20), California (Z intron: *n* = 42; autosomal intron: *n* = 15; ddRADs: *n* = 6) and for the Holarctic region (Z intron: *n* = 87; autosomal intron: *n* = 42; ddRADs: *n* = 21). Where possible the same specimen was sampled for mtDNA, intron and ddRAD data sets, however, this was not always feasible owing to variation in DNA quality and sequencing success. For further details of specimen localities, collection year and which individuals are sequenced in each data set, see Supplementary Data 1.

### Sanger sequencing of mtDNA and nuclear introns

DNA was extracted from frozen tissues and blood samples using DNeasy Tissue Extraction Kits (QIAGEN) using standard manufacturer protocols, and from toe-pads of museum specimens using a modified protocol to increase the yield obtained from these degraded tissue sources. Specifically, 25 μl of DTT (dithiothreitol) solution and 20 μl of Proteinase K were added at the initial digestion step, and at the final step DNA was eluted with 60 μl of elution buffer instead of 200 μl. PCR and sequencing of modern samples followed standard protocols detailed in previous studies of mtDNA^18^ and nuclear introns^39^. PCR of toe-pad samples followed a slightly modified protocol involving increasing template DNA to 3 μl, and adding 4.5 μl of BSA to PCR reactions for samples that failed initial PCR amplification. MtDNA control region was amplified in a single fragment from modern samples and high-quality DNA extractions from toe-pad samples using *Corvus*-specific primers (corII-LGL2, cor-H417^40^), and from more degraded toe-pad samples in two overlapping fragments using additional internal primers designed for this study from representatives of the California, Holarctic and Chihuahuan mtDNA lineages. Primer details were as follows: Fragment 1 (253 bp)—corII-LGL2/cor-H253 (5′-TGGGATTGAGAATTCATTGGRGT-3′); Fragment 2 (147 bp)—cor-L166 (5′-ACAAGACARGCTTCACCCRAG-3′)/cor-H417. Eight nuclear introns were sequenced from seven different chromosomes (chr) using previously published primers^41,42^—chr Z: *ACO1* intron 9; chr 1: *MYO2/MB* intron 2; chr 2: *VIM* intron 8; chr 4: *IRF2* intron 2, *CLOCK* intron 10; chr 6: *PCBD* intron 3; chr 8: *RPL5* intron 4; chr 12: *RHOD* intron 1. DNA extraction and PCR of toe-pad samples were performed in a laboratory free of modern bird DNA or PCR amplicons in order to limit the risk of contamination. All sequences derived from historical museum specimens shared haplotypes with individuals sequenced from contemporary tissues, and did not show other evidence of contamination.

Heterozygous positions in nuclear introns were coded with International Union of Pure and Applied Chemistry (IUPAC) ambiguity symbols. The gametic phase of samples with heterozygous sites were inferred statistically using PHASE v2.1^43^ (five independent runs; −×5 algorithm; 0.70 posterior probability threshold), while the phase of length variant heterozygotes was inferred by the subtraction method (one allele subtracted from the other using chromatograms). Positions with uncertain heterozygous sites (<0.70 posterior probability threshold) were coded with IUPAC symbols. Tests for recombination using the difference of sums-of-squares method were implemented in TOPALi v1^44^ (sliding window 100 bp, 10 bp step size, 0.5 threshold); these tests found no evidence for recombination in the eight nuclear introns examined in this study.

### Library preparation, sequencing and filtering of ddRADs

Double-digest RAD seq (ddRAD) libraries were prepared following Vivian-Smith et al.^45^ (also see Recknagel et al.^46^ for a similar protocol) with minor modifications. Specifically, digestion of the genomic DNA and the ligation of the adaptors were performed in 40 µl in a single step consisting of 1× NEB 4 buffer; 250 µM rATP; 0.5 µM P1 adaptor; 0.5 µM Index A adaptor; 10U SbfI-HF; 10U MspI; 400U T4 ligase; 100–150 ng genomic DNA in a thermal cycler programmed to 37 °C for 1 h; 65 °C for 10 min; slowly cooling to 4 °C (i.e., −1 °C/min to 45 °C; −2 °C/min to 35 °C; −3 °C/min to 15 °C). Between eight and twenty samples were then pooled. After each step in the protocol, the libraries were cleaned with 0.9 volumes of Ampure XP beads (Beckman Coulter) (except after library amplification, see below) and eluted in 0.1× TE-buffer, in an appropriate volume to be used for the next step. Size selection for 430–530 bp fragment sizes was performed on a Blue Pippin (Sage Science), using 2% agarose cassettes and marker V1. Library amplification was performed in 100 µl reactions, consisting of 10U Q5 HiFi polymerase; 1× Q5 reaction buffer; 200 µM of each dNTP; 0.2 µM of each primer and 14 µl template. The reaction conditions were 98 °C for 30 s; (98 °C for 10 s; 60 °C for 15 s; 72 °C for 15 s) x 10 to 14 cycles; 72 °C for 2 min; 4 °C hold. Each reaction was split in four tubes during amplification to reduce PCR duplication. Amplified libraries were cleaned twice using 0.65 volumes of Ampure XP beads to get rid of all short DNA fragments that would inhibit sequencing. The molarity of the clean amplified libraries was detected using the DNF-474 or DNF-488-kits on a Fragment Analyzer (Advanced Analytical). Template preparation of the 400 bp libraries was performed using the Ion One Touch 2 or an Ion Chef (Thermo Fisher Scientific) using 12 or 50 pM of library for the respective systems. Single read sequencing runs were performed on 316v2 and 318v2 chips using Hi-Q chemistry on an Ion Torrent PGM (Personal Genome Sequencer)^47^.

Raw reads were run through Torrent Suite v4.4 from the ion community to filter barcodes and perform default quality screening. Raw reads were exported as FASTQ files, and reads of individuals sequenced across multiple runs were merged into a single FASTQ file. FASTQC (http://www.bioinformatics.babraham.ac.uk/projects/fastqc/) identified adaptor contamination and a drop-off in sequence quality at the end of each read. We therefore used the following pipeline to trim, filter and remove adaptor contamination prior to read mapping: (1) remove adaptor sequences using CUTADAPT v1.9.2^48^ with an error rate of 0.1, a minimum overlap length of 10, and discarding any reads shorter than 50 bp, (2) truncate reads up to the first bp at the 3′ that was above Q17 using FastX-toolkit (http://hannon-lab.cshl.edu/fastx_toolkit/), (3) remove stretches of poly A/Ts from the 3′ end that were at least 4 bp long using PRINSEQ-LITE v0.20.4^49^, and finally (4) FastX-toolkit was used to trim the last 20 bp from all reads, truncate reads to a maximum of 300 bp, remove reads shorter than 50 bp and only keep reads where over 95% of bases had quality scores above Q13 and 80% of bases had quality scores above Q17. Of the 63 specimens initially sequenced, we excluded 10 owing to poor sequence quality indicated by raw reads below 190,000. This resulted in a final data set with 6 Chihuahuan Ravens and 47 Common Ravens of which 21 are from purer Holarctic regions (Alaska, Canada, east United States), 6 are from purer California regions (California) and 20 are from admixed regions in the western United States. For details of per individual raw ddRAD reads, see Supplementary Data 1.

### Filtering and variant calling of ddRADs

Filtered reads were mapped to the genome of the American Crow *Corvus brachyrhynchos*^50^ using the tmapall function in the torrent mapping alignment programme TMAP v4.4 (https://github.com/iontorrent/TS/tree/master/Analysis/TMAP). TMAP optimises a series of established packages (e.g., BWA^51^) for use with Ion Torrent data and outperforms other read mapping programs for these data owing to its ability to better handle alignments of indels caused by the high rate of homopolymer errors typical of Ion Torrent sequencing technology^52^. Variant calling and additional filtering were performed in samtools and BCFtools^53^, which have been shown to perform best for Ion Torrent data, especially when paired with TMAP^52,54^. Our variant calling pipeline had the following steps: (1) reintroduce sample IDs into the BAM file headers produced by TMAP using samtools view, sed and samtools reheader, (2) sort and index BAM files using samtools sort and samtools index, (3) index reference using samtools faidx, (4) run samtools mpileup for all individual BAM files against this reference using a homopolymer coefficient of 50 (-h)^52^, minimum number of 5 reads to call an indel (-m), and applying the -C50 coefficient to better handle short reads with excessive mismatches and (5) call variants using ‘bcftools call’ with the multi-allelic caller and outputting variant sites only. We then performed the following filtering steps using ‘bcftools filter’, SNPSift^55^ and VCFtools^56^: (1) exclude all indels and any SNP within 3 bp of an indel and/or with an average quality score less than Q13, (2) only retain SNPs present in at least five individuals with an average quality score above Q20 and a minimum depth per individual of 5 and a maximum average depth of 500, (3) only retain SNPs with a quality score per individual greater than Q20. We then excluded SNPs that were missing from more than 50% of individuals using vcftools–max-missing. To limit the impact of linkage disequilibrium, we first randomly thinned SNPs that were within 50 bp of each other using VCFtools–thin and then we used PLINK v1.09^57^ to remove SNPs that violated Hardy–Weinberg equilibrium (HWE) and SNPs that were putatively linked based on a squared coefficient of correlation threshold in excess of *R*^2^ = 0.8 using a window of 1 kb. We also exported an additional SNP data set with less-stringent filtering in order to explore the genomic landscape of divergence (see below). This data set allowed 30% missing data and did not exclude loci based on linkage disequilibrium (LD), HWE or proximity on scaffolds in the *C*. *brachyrhynchos* genome.

The final stringently filtered SNP data set was exported from PLINK allowing for SNPs to be missing from either 10, 30 or 50% of individuals, and these were calculated separately for data sets containing all Common Ravens with or without Chihuahuan Ravens. For the data set containing only Common Ravens (*n* = 47), this resulted in 1233–2301 SNPs depending on the amount of missing data allowed (10% missing: 1233 SNPs, overall genotyping rate = 0.982; 30% missing: 1838 SNPs, genotyping rate = 0.923; 50% missing: 2301 SNPs, genotyping rate = 0.857). The data set containing both Common (*n* = 47) and Chihuahuan (*n* = 6) Ravens resulted in 1205–2205 SNPs (10% missing: 1205 SNPs, genotyping rate = 0.979; 30% missing: 1737 SNPs, 0.926 genotyping rate; 50% missing: 2205 SNPs, genotyping rate = 0.851). Although acknowledging that including more SNPs despite missing data generally increases the power of analyses, we chose to use stringent filters on data sets to be used for population genetic and phylogenetic analyses in order to reduce the impact of known issues with Ion Torrent data^46,52,54^.

### Delineating raven lineages and geographic structure

For mtDNA, we estimated a maximum likelihood (ML) phylogeny in RAXML v8.0^58^ from 313 bp alignment of mtDNA control region. We included sequences from GenBank for six other species representing the major clades in *Corvus*^59^ (*C*. *albus*, *C*. *albicollis*, *C*. *brachyrhynchos*, *C*. *frugilegus*, *C*. *hawaiiensis*, *C*. *kubaryi*), and used the more distantly related *Nucifraga columbiana* as an outgroup. RAXML analyses used the ‘fast ML’ algorithm with 100 bootstrap pseudoreplicates and the GTRCAT substitution model. Phylogeographic structuring within each mtDNA lineage was explored using unrooted parsimony networks estimated in TCS v1.21^60^ with 95% connection limit from mtDNA alignment that was trimmed to 254 bp and excluded 26 incomplete sequences in order to reduce the impact of missing data. Samples from *C*. *c*. *tingitanus*—a genetically distinct raven subspecies from the Canary Islands off the coast of northwestern Africa^24^—were omitted from the Holarctic mtDNA network since they did not connect to other Holarctic clade samples under a 95% connection limit.

For nuclear introns, we estimated unrooted parsimony networks in TCS v1.21^60^ with 95% connection limit using phased sequences from each nuclear intron, and then used Bayesian clustering in STRUCTURE 2.3.3^61^ and unrooted Neighbour-Net trees in SplitsTree v4.14.2^62^ to infer geographic structuring and delineate major lineages across all seven autosomal introns. STRUCTURE analyses used unique haplotypes rather than nucleotides in order to avoid biases associated with using multiple linked SNPs within each locus as input data. Preliminary runs for genetic clusters (K) ranging from 1 to 6 showed that the data had little information about population structuring after K3. Final analyses were run ten times for each genetic cluster between 1 and 4 with an initial burnin period of 50,000 generations and 500,000 MCMC generations under an admixture model with correlated allele frequencies among populations. We then used CLUMPAK^63^ to select the best value of K using the Evanno Delta K method and to summarise the ten independent runs from each K under default parameters. For Neighbour-Net trees, unphased sequences from the seven autosomal introns with heterozygous sites coded with IUPAC symbols were concatenated for each individual. Neighbour-Net tree analyses used uncorrected *P* distances and considered heterozygous sites as average states.

For genome-wide ddRAD SNPs, we used smartPCA (principal component analysis) in the EIGENSOFT package^64^, Bayesian clustering in STRUCTURE and unrooted Neighbour-Net trees in SplitsTree to infer geographic structuring and delineate major lineages in data sets containing either all ravens (Common *n* = 47, Chihuahuan *n* = 6; K 1–4) or only Common Ravens (*n* = 47; K 1–3) and with differing amounts of missing data (10, 30 and 50%). Neighbour-Net and STRUCTURE analyses were performed as per the autosomal introns. Results were near-identical for data sets containing 10% (all ravens: *n* = 1205 SNPs; Common Ravens only: *n* = 1233 SNPs), 30% (all ravens: *n* = 1737 SNPs; Common Ravens only: *n* = 1838 SNPs) and 50% (all ravens: *n* = 2205 SNPs; Common Ravens only: *n* = 2301 SNPs) missing data. We therefore show PCA and STRUCTURE results for the 10 and 50% missing data sets, and Neighbour-Net trees for the 10% missing data set.

### Genomic landscape of lineage fusion

Measures of population differentiation (*F*_ST_: autosomal introns and SNPs; *ϕ*_ST_: mtDNA and Z intron) and genetic diversity (mtDNA and Z intron: nucleotide diversity (*π*); autosomal introns and SNPs: *H*_o_/*H*_e_) were estimated for Chihuahuan Ravens, Common Ravens, California lineage, Holarctic lineage and the admixed western USA region based on the mtDNA, Z intron, autosomal intron and SNP (stringently filtered 10% missing) data sets, and for the seven autosomal introns independently using a locus-by-locus AMOVA in Arlequin v3.5.1.2^65^ with 100 permutations. To explore genomic landscape, we used PLINK v1.09^57^ to estimate Wright’s *F*_ST_ (Weir and Cockerham’s method) between each lineage at each SNP. We then plotted *F*_ST_ between each pair of lineages for each SNP against their position on scaffolds in the *C*. *brachyrhynchos* reference genome using the less-filtered data set (30% missing data, no LD or distance filtering) with and without excluding SNPs that violated HWE. Putatively admixed individuals from Canada were not included in the Holarctic lineage for these measures of *F*_ST_. SNPs within the top 5% of *F*_ST_ values between each pair of lineages in the stringently filtered data set (10% missing data; LD and HWE filters) and SNPs with *F*_ST_ > 0.2 between each pair of lineages in the less-filtered data set (30% missing data; no HWE filtering) were exported for phylogenetic analyses and to create individual genomic profiles (see below). To limit the impact of potentially linked SNPs on these analyses, we excluded SNPs in the less-filtered data set that were within 5000 bp of each other.

### Characterising admixture and mosaic genomes

To test for a signal of admixture between lineages, we used three-population test f3 statistics as implemented in TreeMix v1.12^27,28^ on the 10% missing SNP data set with windows of 500 SNPs. We considered pairwise comparisons between either California, Holarctic and western United States or California, Holarctic and Chihuahuan Ravens. Significantly negative f3 statistics and *Z* scores from three-population tests provide strong support for substantial admixture.

To explore the geographic pattern of admixture, we plotted the average proportion of Holarctic ancestry for 86 populations in North America against increasing distance from San Diego, California (calculated ‘as-the-crow-flies’). To aid interpretability, these graphs exclude populations from the eastern United States and Mexico. Populations were designated by grouping geographically proximate specimens within a state or province. For Z intron and mtDNA data sets, ancestry proportions represent the observed frequency of California and Holarctic mtDNA lineages or *ACO1* California/Holarctic SNP for each population. For autosomal introns and ddRAD SNPs, ancestry proportions represent STRUCTURE assignments to Holarctic and California genetic clusters averaged across individuals at each population.

To explore evidence for mosaic genomes and test the hypothesis of no assortative mating between California and Holarctic lineages^20^, we created genomic profiles for all Common Ravens that were sampled in at least three data sets by scoring each individual as belonging to Holarctic, California or admixed genetic clusters in each data set. For mtDNA, individuals were assigned to Holarctic and California lineages using RAXML. For Z-linked intron, individuals were assigned to California and Holarctic lineages based on the A/G SNP that separated California alleles and a single Holarctic allele in the unrooted network. Heterozygotes for this SNP were assigned as ‘admixed’. For autosomal introns and ddRAD SNPs, individuals were assigned to California and Holarctic lineages based on their STRUCTURE membership coefficients (membership coefficients above 0.7 for either Holarctic or California lineages were assigned as ‘pure’, while those with coefficients between 0.3 and 0.7 were assigned as ‘admixed’).

To further explore whether Common Ravens in admixed regions show evidence of having substantially admixed nuclear genomes that are a mosaic of California and Holarctic parental lineages, we created SNP-level genomic profiles. We selected all SNPs from the less-filtered data set (30% missing data; no HWE filtering; SNPs within 5000 bp of each other excluded) that were fixed (*F*_ST_ = 1, *n* = 5 SNPs) or almost fixed (*F*_ST_ > 0.58; *n* = 20 SNPs) between pure Holarctic and California lineages. For each bi-allelic SNP, we designated the allele that was fixed or near-fixed in the pure populations as California or Holarctic and then we scored each individual from the rest of the range as homozygous or heterozygous for these California or Holarctic alleles at each SNP. If mosaic genomes exist and gene flow is unrestricted between California and Holarctic lineages in the admixed western United States populations, we expect to find uncoupling of mtDNA and nuclear assignments (i.e., western USA individuals with California or Holarctic mtDNA may have any proportion of California/Holarctic nuclear ancestry) and we expect to see evidence of admixture even in SNPs that are fixed and nearly fixed between pure California and Holarctic regions (i.e., western USA individuals will have high proportions of heterozygous SNPs and/or a mixture of homozygous California and Holarctic SNPs).

### Exploring discordant phylogenetic signals

The phylogenetic signal in the nuclear genome was explored using Neighbour-Net and species tree approaches. Three data sets were examined: (1) seven autosomal introns, (2) all genome-wide SNPs (1175 SNPs from the stringently filtered data set allowing 10% missing data) and (3) SNPs with the highest *F*_ST_ values between California and Holarctic lineages. Species tree analyses based on SNPs with the highest 5% of *F*_ST_ values from the stringently filtered data set (10% missing data; LD and HWE filters applied; *n* = 60 SNPs) failed to converge. Instead, we use SNPs with *F*_ST_ > 0.2 from the less-filtered data set (30% missing data; no HWE filtering and SNPs within 5000 bp of each other excluded; *n* = 131 SNPs; mean *F*_ST_ = 0.35). These high *F*_ST_ SNPs are potentially resistant to admixture between collapsing California and Holarctic lineages, and thus our goal was to test whether phylogenetic analyses of these SNPs support the same topology as mtDNA or whether they support the same topology as the other nuclear data sets. For comparison we also estimated Neighbour-Net trees for SNPs from the less-filtered data set with *F*_ST_ > 0.2 between California and Chihuahuan Ravens (*n* = 315 SNPs; mean *F*_ST_ = 0.43) and Holarctic and Chihuahuan Ravens (*n* = 309 SNPs; mean *F*_ST_= 0.47). The stringently filtered and less-filtered SNP data sets produced near-identical patterns of population structure in all four subsets of SNPs examined (Supplementary Fig. 4). This indicates that our exclusive use of the less-filtered SNP data set for species tree analyses of high *F*_ST_ SNPs between the California and Holarctic lineages should not have biased our inferences about topologies from high *F*_ST_ SNPs (Fig. 5c).

For each data set, we used unrooted Neighbour-Net trees in SplitsTree v4.14.2^62^ and smartPCA in the EIGENSOFT package^64^ to explore the different phylogenetic signals present in each of data set. In all analyses, admixed individuals from the western United States connected distinct California and Holarctic lineages based on pure individuals alone (Supplementary Figs. 2 and 4). Since hybridisation violates the models implemented in *BEAST and SNAPP, species trees were only estimated using pure individuals that showed the least signal of admixture (Holarctic: all individuals from ME, NJ, NY; California: all individuals from CA except CA899 for SNPs and CAIS1, CAIS2, CAIS3, CAIS4, CA895, CA881 and NCA56 for autosomal introns). We excluded all individuals from the highly admixed populations in the western United States, as well as Holarctic individuals from the northern and eastern United States (Alaska, Minnesota, Michigan and Wisconsin) and Canada that showed a signal of introgression of California alleles in different data sets. Owing to issues with reaching convergence for computationally intensive SNAPP analyses, we further reduced the SNAPP analyses to two individuals for each pure lineage (California: CA1714, CA887; Holarctic: ME838, ME850; Chihuahua: CY18, CY21) (see Supplementary Data 1 for sample details).

Unrooted Neighbour-Net analyses in SplitsTree v4.14.2^62^ used uncorrected *P* distances and average states for heterozygous sites. Species trees were estimated in BEAST v2.4.3^66^ using *BEAST^67^ for the autosomal introns and SNAPP^68^ for the SNP data sets. *BEAST species tree analyses on the autosomal intron data set used a Yule speciation prior and applied a lognormal prior on birthrate (*M* = 4.0, *S* = 1.25) and population mean (*M* = 5.0, *S* = 1.2). We applied a strict clock for all nuclear loci and used an exponential prior on the clock rate for all loci (*M* = 1.0), except for VIM intron 8, which was fixed to 1.0. HKY + I + G was used as the substitution model on all nuclear loci and we used empirical base frequencies, estimated kappa, gamma, shape and proportion of invariants. *BEAST was run twice for 1 × 10^8^ generations, sampling every 5000 generations and with a burnin of 1 × 10^7^ generations. SNAPP species tree analyses on the SNP data sets used a Yule prior for the species tree, a 1/X hyper prior on lamda and default settings for all other priors. We calculated mutation rate U and V for each data set using BEAUti 2, these were fixed in all analyses while coalescent rate was estimated (initial value = 10). SNAPP was run twice for 1 × 10^7^ generations, sampling every 1000 generations and with a burnin of 1 × 10^6^ generations. For both *BEAST and SNAPP analyses, we used TRACER v1.6 (http://tree.bio.ed.ac.uk/software/tracer/) to ensure convergence of independent runs (as indicated by stationary posterior and prior traces, high ESS and near-identical marginal probability estimate distributions on all parameters), and then used LOGCOMBINER to combine log and tree files from the two independent runs after excluding burnin. TREEANNOTATOR was used to estimate the maximum clade-credibility tree (MCC) using mean heights for each data set. Finally, TreeSetAnalyser was used to calculate the 95% HPD of tree topologies and the percentage of trees within this 95% HPD set that supported a sister relationship between California and Holarctic lineages, California and Chihuahuan lineages or Holarctic and Chihuahuan lineages.

### Timing mtDNA divergences

Previous divergence estimates for the North American raven lineages were not tree-based, and therefore do not account for possible rate heterogeneity across lineages^19^. We therefore used BEAST v1.7.5^69^ to more robustly estimate the timing of divergence of mtDNA lineages. We collapsed mtDNA sequences from 441 Common Ravens and 28 Chihuahuan Ravens into 143 unique haplotypes and included *C*. *brachyrhynchos* MSB20931 as an outgroup. JModeltest2^70^ was used to select the best substitution model for this data set using default parameters, a BioNJ starting tree and the AICc criteria to select the best substitution model. BEAST analyses used a Yule speciation prior on the tree model, HKY + G substitution model as selected by JModeltest2, empirical base frequencies and an uncorrelated lognormal relaxed clock with a lognormal prior with a mean of 0.026 and log standard deviation of 0.12 on the substitution rate. This prior distribution captured the substitution rate inferred for control region from the 95% HPD interval estimated in Lerner et al.^71^ (0.019–0.035 substitution/site/lineage/mya). BEAST analyses were run twice for 1 × 10^7^ generations sampling every 1000 generations with a burnin of 1 × 10^6^ generations. Independent runs were combined, and final MCC trees calculated using the same approach as per *BEAST and SNAPP species tree analyses for nuclear data sets.

### Testing the suitability of Pleistocene climates

Maximum entropy species distribution modelling in MAXENT v3.3.3.k^31^ was used to predict ecological niche models (ENMs) using bioclimatic variables with 2.5 arc-min resolution from the WorldClim database^72^ and geo-referenced occurrence records for Common Ravens and Chihuahuan Ravens obtained from GBIF (Global Biodiversity Information Facility; http://www.gbif.org/) combined with geo-referenced localities of genomic samples used in this study (see Supplementary Data 1 and Supplementary Fig. 5). Species locality data were verified and refined according to species ranges^29^. ENMtools^73^ was used to remove duplicates and records that fell within the same grid cell in order to avoid overfitting ENMs. This resulted in a data set of over 90,000 localities for Common Ravens, and 5000 localities for Chihuahuan Ravens. In addition to performing ENMs from all Common Raven localities, we also used subsets of the data set to explore whether regions with purer California ancestry (California, Arizona and Mexico), purer Holarctic ancestry (Canada, east United States and Alaska) and admixed ancestry (all western United States populations except Arizona) are predicted to have disjunct or overlapping distributions during the Pleistocene. We used the following regional subsets in the absence of mtDNA genotypes for all GBIF occurrence records: (1) separate ENMs for GBIF samples from regions with purer California, purer Holarctic and admixed ancestry, (2) single ENM combining GBIF samples from purer Holarctic and admixed western United States populations, (3) single ENM combining GBIF samples from purer California and admixed western United States populations and (4) separate ENMs using only individuals genotyped as having either Holarctic or California mtDNA.

The 19 bioclimatic variables were cropped to North America, and then ENMtools was used to test for correlations between bioclimatic variables. Final present-day ENMs were predicted using ten bioclimatic variables that were not strongly correlated (Pearson’s correlation <0.9: bio2, bio5, bio6, bio7, bio8, bio12, bio15, bio17, bio18, bio19) under recommended default settings (regularisation = 1, iterations = 500, convergence threshold = 0.00001) and using a random 30% of samples for testing following recommendations in ref. ^74^. Paleo-ENMs were predicted by projecting these present-day ENMs onto bioclimatic variables predicted under both the community climate system model (CCSM)^75,76^ and the model for interdisciplinary research on climate model (MIROC-ESM)^77^ for climatic conditions during the last glacial maximum (LGM, 18–21 ka)^78^ and with ‘fade-by-clamping’ selected^74^. MIROC and CCSM models differ in their estimates of precipitation and temperature for the LGM, and thus their combined use offers a better estimate of putative suitable conditions for the species being modelled^66–69^. Model fit and accuracy was evaluated by visually comparing present-day ENMs to species range limits^29^, and using area under the receiver operating curve measures (AUC). Note that AUC scores for widespread generalist species with extensive presence records, like Common Ravens, are expected to be lower than those with highly restricted ranges, where an AUC of 1.0 is considered to indicate good fit^30^.

### Data availability

British Columbia specimens collected for this study are accessioned at the UBC Beaty Biodiversity Museum-Cowan Tetrapod Collection. Sanger sequences were deposited in GenBank under the following accession numbers—mtDNA control region: MG594926–MG595149; *ACO1*: MG591008–MG591247; *RHOD*: MG583407–MG583517; *IRF2*: MG590382–MG590504; *PCBD*: MG590505–MG590618; *MYO2/MB*: MG590619–MG590732; *RPL5*: MG590733–MG590880; *VIM*: MG590881–MG591007; *CLOCK*: MG583286–MG583406. Raw ddRAD reads were deposited in sequence read archive under BioSample accession numbers SAMN08118047–SAMN08118108.

## Electronic supplementary material

Supplementary Information

Description of Additional Supplementary Files

Supplementary Data 1
