# Supplementary material for: Genomic evidence of speciation reversal in ravens
Source: Nat Commun. 2018 Mar 2;9:906. doi: 10.1038/s41467-018-03294-w (PMC5834606; doi:10.1038/s41467-018-03294-w)
Supplement: Supplementary file 1 — Supplementary Information [file 41467_2018_3294_MOESM1_ESM.pdf]

SUPPLEMENTARY INFORMATION

Kearns et al.

Genomic evidence of speciation reversal in ravens

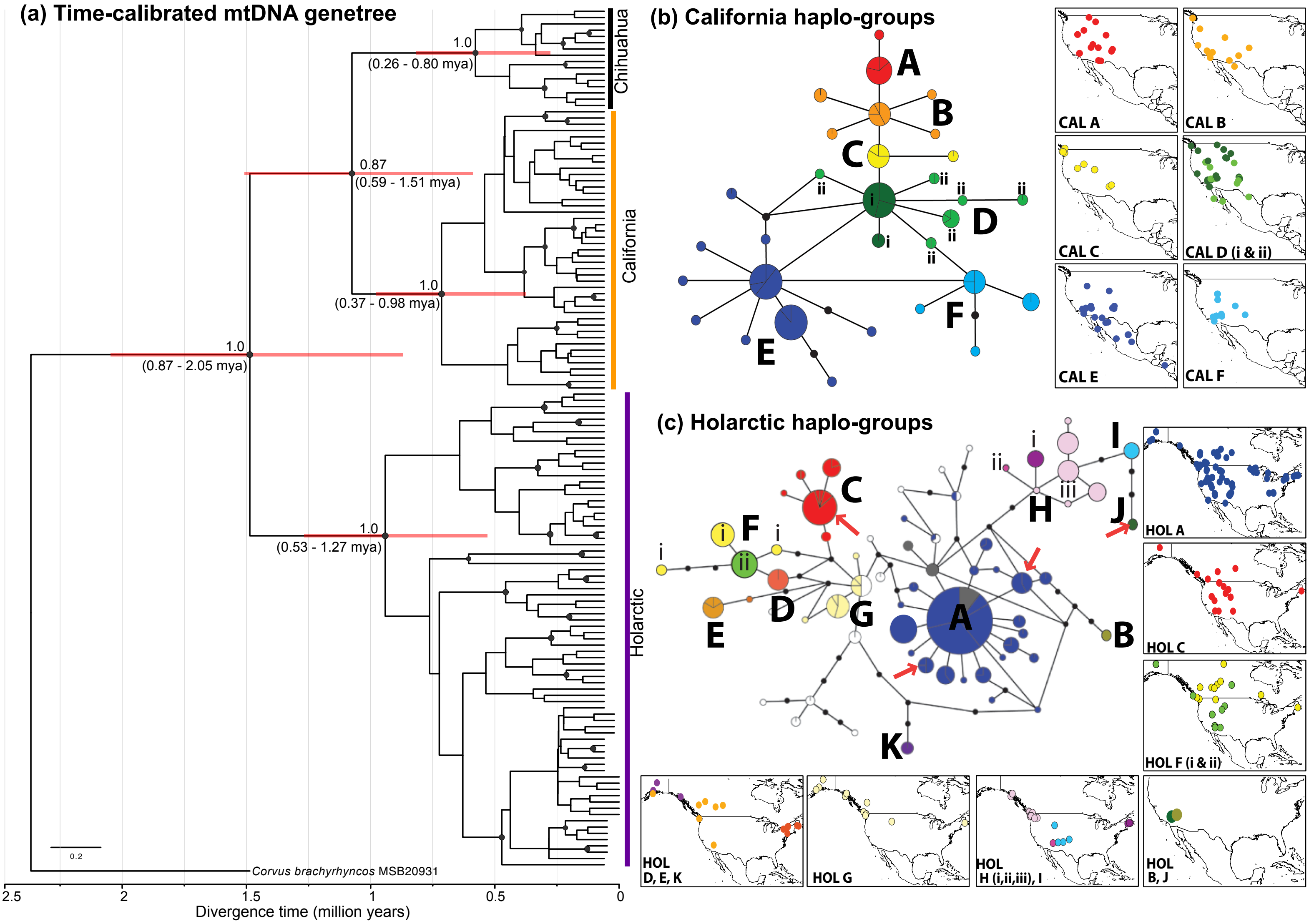

Supplementary Figure 1. Geographic structuring of Holarctic and California lineages of Common Ravens inferred from mtDNA . (a) Time-calibrated mtDNA gene tree for the North American ravens. Chronogram shows the maximum-clade-credibility tree obtained from two independent MCMC chains. Posterior probability support above 0.5 is indicated with a black circle on the node, and support values are given for five key nodes in the phylogeny. The 95% highest posterior density range for divergence time estimates of key nodes are indicated with horizontal node bars and ranges in brackets. Unrooted network of mtDNA control region for (b) California and (c) Holarctic mtDNA lineages. Coloured circles represent unique mtDNA haplotypes scaled by sampling frequency. Small black circles represent unsampled haplotypes. Lines between each circle represent a 1 bp difference between connected haplotypes. Red arrows in (c) show the four Holarctic mtDNA haplotypes found in the state of California. Maps show the geographic distribution of major haplo-groups or key haplotypes within Holarctic and California mtDNA lineages with colours corresponding to haplotypes in (b) and (c) (samples from Old World (white) and Greenland (dark grey) are not shown on maps).

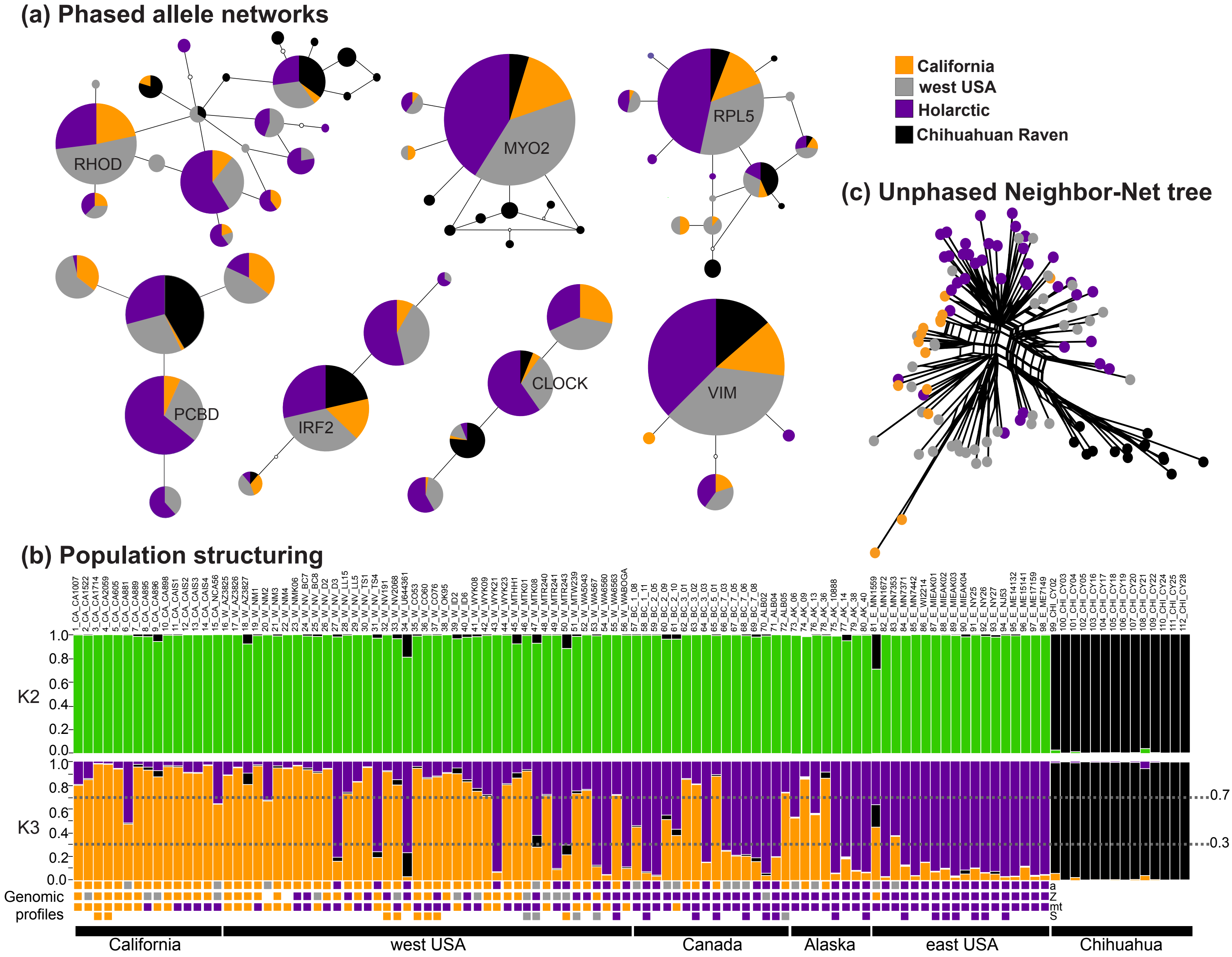

Supplementary Figure 2. Geographic structuring of Holarctic and California lineages of Common Ravens inferred from seven autosomal introns. (a) Unrooted allele networks for each intron. Pie-graphs representing unique alleles are scaled by sample size with colours corresponding to Chihuahuan Ravens (black) and Common Ravens from regions with pure California (orange; sample size per state/province = CA: 15), pure Holarctic from Canada, Alaska and eastern US (purple; sample size per state/province = AK: 8, BC: 13, ALB: 3, WI: 1, MN: 5, MI:4, NY: 3, NJ: 1, ME:4) and admixed western US (grey; sample size per state = WA: 5, MT: 7, WY: 4, CO: 3, OK: 1, NM: 5, AZ: 3, NV: UT: 1, NV: 10, ID: 2) ancestry. Small white circles in networks represent unsampled putative alleles. (b) STRUCTURE plots depicting the probability of assignment of each individual to a population under a model assuming either two (K2) or three (K3) populations. Two populations was selected as the best fit for the data (Delta K/mean LnP(K): K2 = 1183.3/ -1652.2, K3 = 85.6/-1606.2). Common Ravens are sorted by increasing distance from southern California through to Canada, Alaska and east US. Dashed lines indicate the threshold used to assign Common Ravens to California (> 0.7 orange cluster), Holarctic (< 0.3 orange cluster) or admixed (0.3-0.7 orange cluster) genetic clusters. Squares below each vertical bar in the STRUCTURE plots show the assignment of individuals based on this analysis of autosomal introns, and for Z intron, mtDNA and ddRAD SNPs (acronyms: a, Z, mt, S). (c) Unrooted Neighbor-Net trees from unphased concatenated autosomal introns.

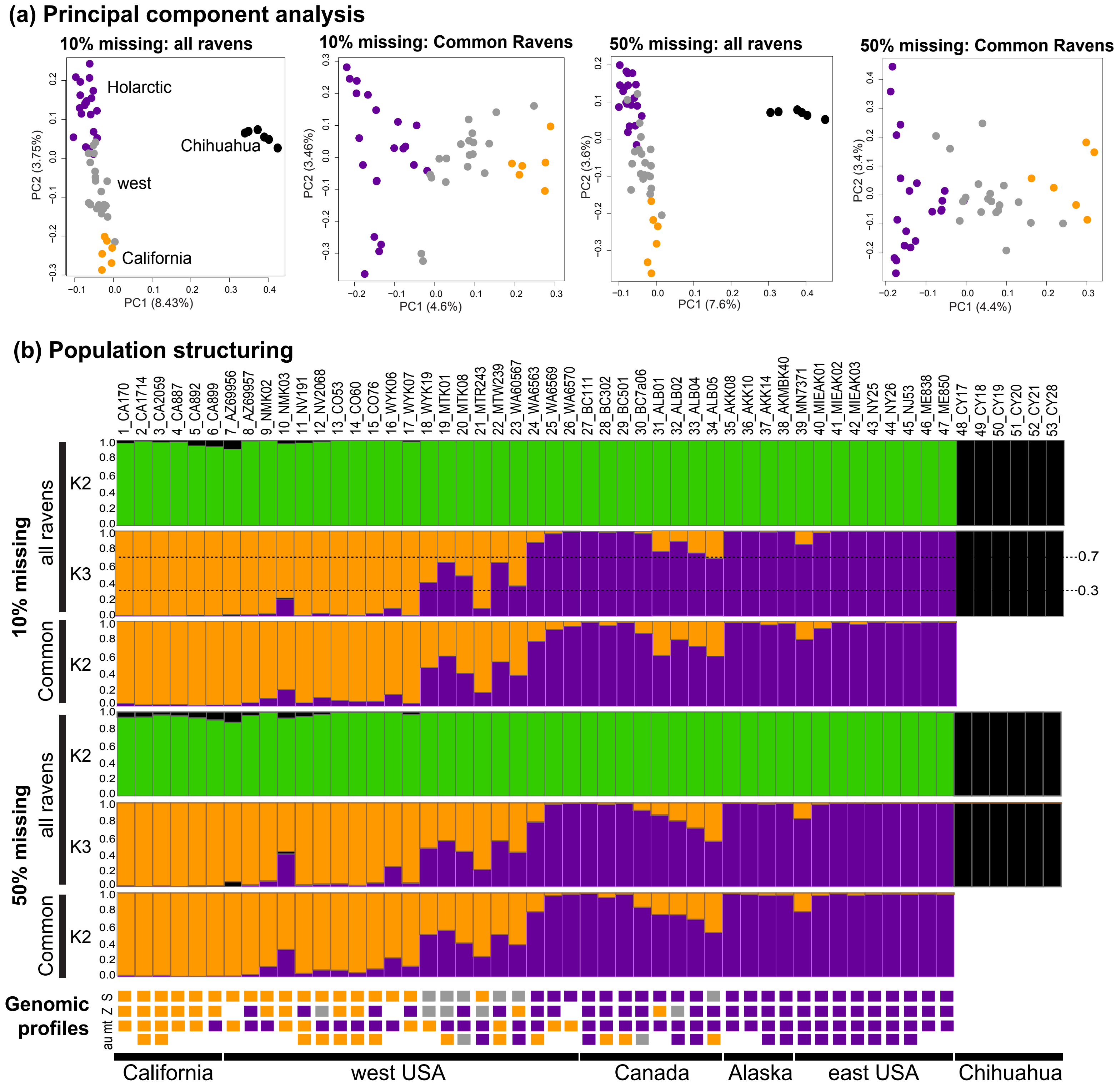

Supplementary Figure 3. Geographic structuring of Holarctic and California lineages of Common Ravens inferred from genome-wide SNPs. (a) Principal component analysis (PCA) showing discrete clusters for Chihuahuan and Common Ravens from 'pure' Holarctic (northern and eastern North America) and 'pure' California (California) regions that are connected by individuals from admixed populations in the western US. Circles represent PC1 and PC2 scores for each individual with colours denoting samples from Chihuahuan Ravens (black) and Common Ravens from regions with pure California (orange), pure Holarctic (purple) and admixed (grey) ancestry. (b) STRUCTURE plots depicting population assignment of Common Ravens and Chihuahuan Ravens under two (K2) and three (K3) putative populations for datasets including all ravens or only Common Ravens with either 10% or 50% missing data allowed. Each bar represents the probability of assignment of each individual to a population given a particular K. Common Ravens are sorted by increasing distance from southern California through to Canada, Alaska and east US. Dashed lines show the threshold used to assign individuals to California, Holarctic or admixed clusters as per Supplementary Figure 2. Squares below each vertical bar show the assignment of individuals to each lineage based on this analysis of ddRAD SNPs, and for Z intron, mtDNA and autosomal introns (acronyms: S, Z, mt, au). Two populations was selected as the best fit for the data: Delta K/mean LnP(K): 10% missing K2 =1389.9/-47432.7, K3 = 17.3/-47169.9; 30% missing: K2 =551.2/-63879.6, K3 = 91.7/-63460.5; 50% missing: K2 =737.3/-76251.2, K3 = 548.5/-75799.9.

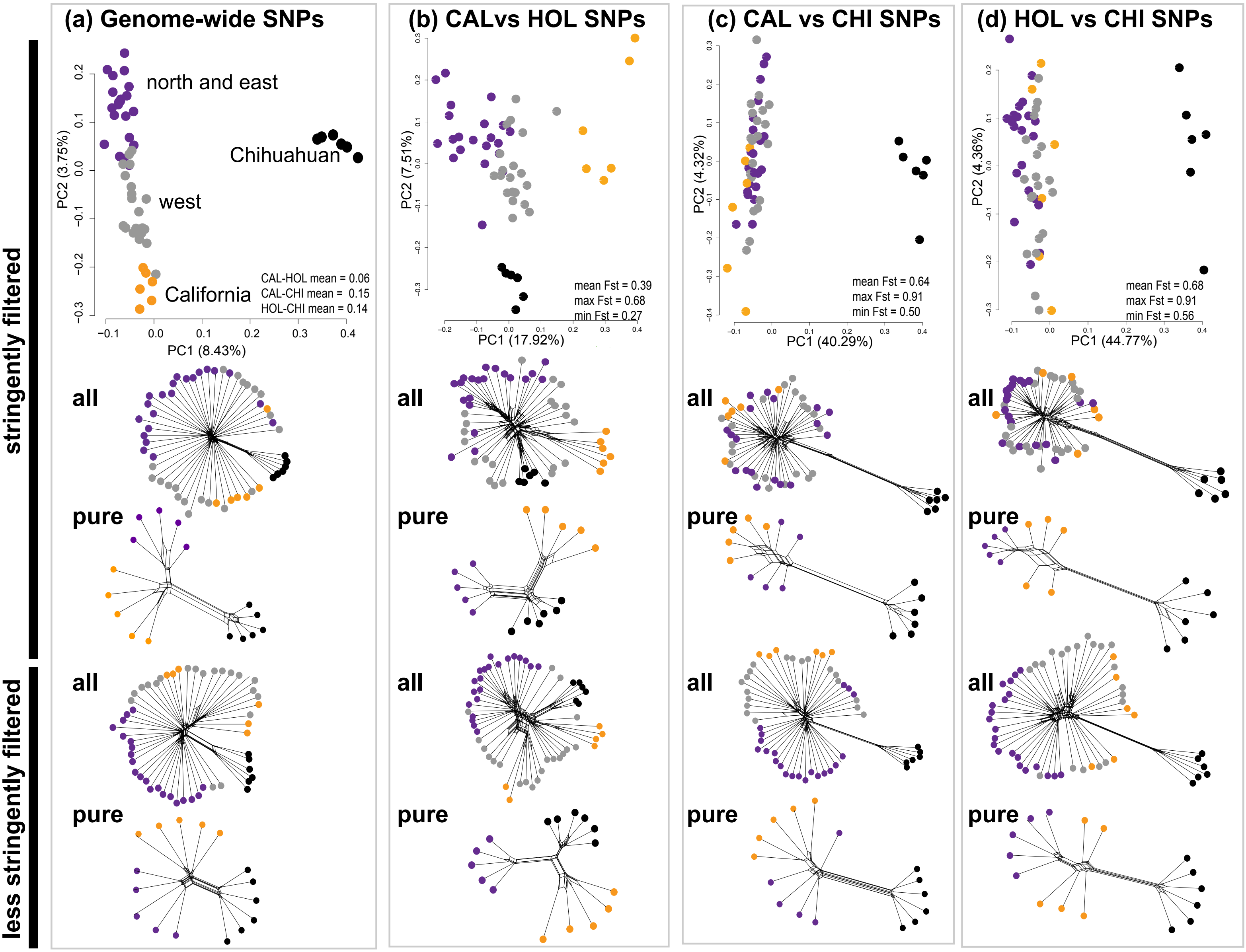

Supplementary Figure 4. Phylogenetic relationships inferred from four subsets of SNPs. (a) all genome-wide SNPs, (b) high *F*st SNPs between California and Holarctic lineages, (c) high *F*st SNPs between California and Chihuahuan lineages, and (d) high *F*st SNPs between Holarctic and Chihuahuan lineages. For each panel we show a PCA from the stringently filtered dataset (10% missing data with LD and HWE filtering), and unrooted Neighbor-net phylogenies from the stringently filtered dataset and the less-stringently filtereed dataset (30% missing data without LD and HWE filtering) including either all pure and admixed individuals or only including pure individuals that showed the least signal of admixture (California: all CA individuals except CA899; Holarctic: all individuals from ME, NJ and NY; Chihuahuan: all individuals). High *F*st SNPs represent SNPs with the top 5% of *F*st values for the stringently filtered dataset (n = 60 for all pairs) and all SNPs with *F*st > 0.2 between each pair of lineages for the less-stringently filtered dataset (n = 131 for CAL vs HOL; n = 315 for CAL vs CHI; n = 309 for HOL vs CHI). Colours follow previous Figures: Chihuahuan Ravens (CHI, black), Holarctic (HOL, purple) and California (CAL, orange).

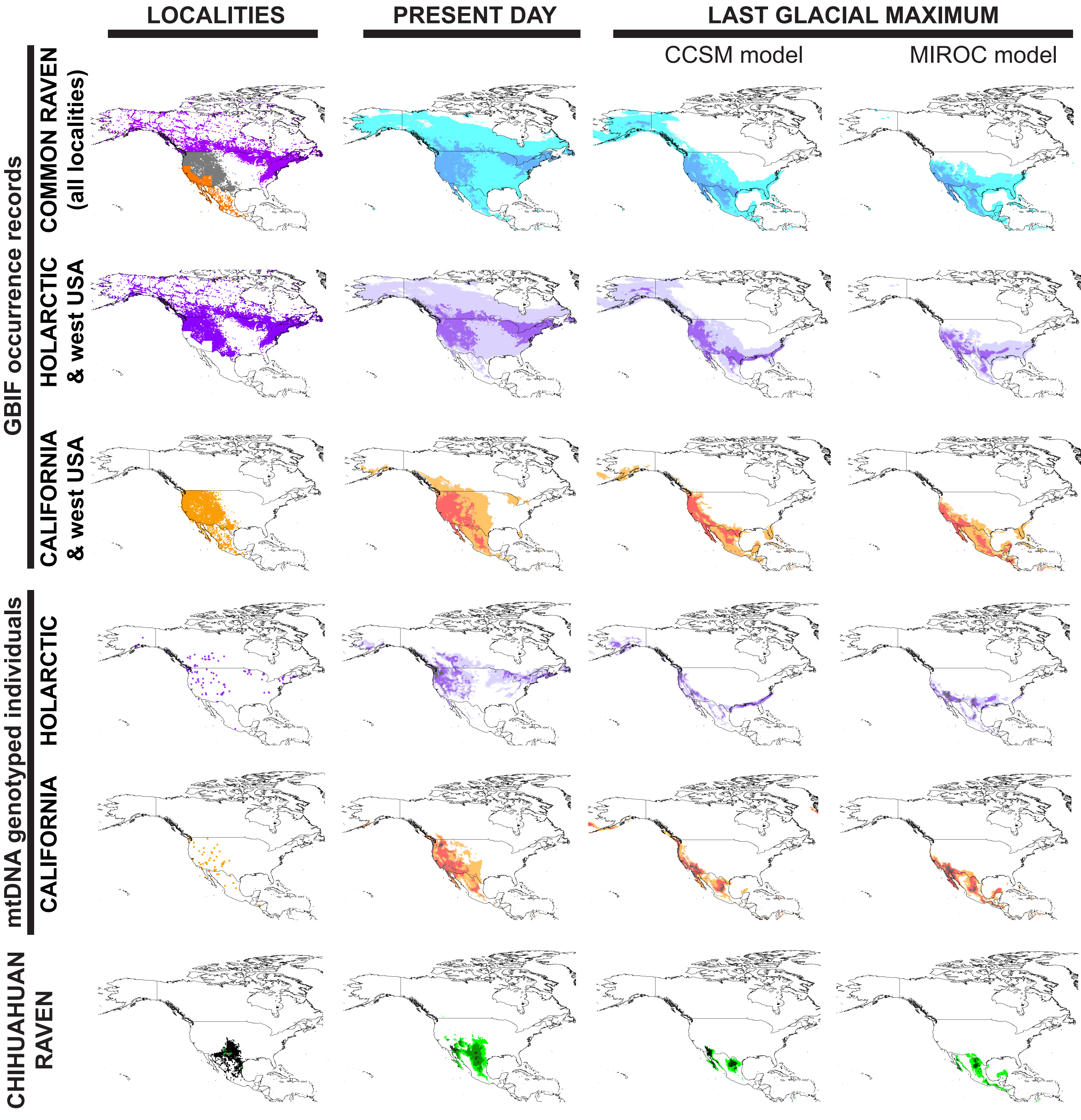

Supplementary Figure 5. ENMs for North American raven lineages under present-day and Pleistocene LGM (18 - 21 kya) climatic conditions. Different subsets of occurrence records predict similar ENMs for each lineage, which show an overlap in suitable climatic niches in western US and Mexico for Holarctic, California and Chihuahuan Raven lineages during the LGM under both MIROC and CCSM paleo-climate models. Relative climatic suitability is divided into four bins (0 - 0.25, 0.25 - 0.5, 0.5 - 0.75, 0.75 - 1.0). Regions with lowest suitability (< 0.25) are shown in white, and increasing darkness of colours indicates increasingly suitable conditions. Test AUC scores for each model followed by the maximum possible test AUC score in brackets and the standard deviation are as follows for each set of occurrence records examined: All Common Raven localities from GBIF 0.57 (0.58) ± 0.002; Holarctic plus admixed western US regions from GBIF 0.59 (0.59) ± 0.008; California plus admixed western US regions from GBIF 0.65 (0.66) ± 0.003; Individuals with Holarctic mtDNA 0.89 (0.90) ± 0.023; Individuals with California mtDNA 0.95 (0.94) ± 0.008; Individuals with Chihuahuan mtDNA 0.99 (0.97) ± 0.001. GBIF occurrence records for Common Ravens shown in the top left map are colour coded by regions used in ENMs shown in Figure 6 for California (orange), Holarctic (purple) and admixed western US (grey). GBIF occurrence records used in Figure 6 for Chihuahuan Ravens are shown in black in the bottom left map along with geo-referenced localities (in green) for Chihuahuan Ravens whose mtDNA was sequenced for this study and used to predict the ENMs based on mtDNA genotyped individuals (bottom panel).

Supplementary Table 1. **Mean pairwise F_{ST}/φ_{ST} estimates for mtDNA, Z intron, autosomal introns, and ddRAD SNPs.**

	mtDNA	Z intron	autosomal introns	All SNPs	Top 5% SNPs
Chihuahuan vs Common	0.54	0.70	0.31 (0.03-0.43)	0.17	0.71 (0.55-0.91)
Chihuahuan vs Holarctic	0.75	0.94	0.36 (0.02-0.46)	0.20	0.7 (0.57-0.87)
Chihuahuan vs California	0.66	0.79	0.41 (0.02-0.46)	0.22	0.64 (0.49-0.91)
California vs Holarctic	0.59	0.93	0.15 (0.02-0.46)	0.04	0.39 (0.28-0.68)
California vs west US	0.13	0.38	0.04 (0.02-0.46)	0.01	0.28 (0.18-0.50)
Holarctic vs west US	0.25	0.51	0.04 (0.02-0.46)	0.02	0.17 (0.12-0.32)

F_{ST} for the seven autosomal introns is presented with the mean and range of F_{ST} estimates from individual loci in parentheses. F_{ST} for SNPs is estimated across all SNPs in the 10% missing stringently filtered SNP dataset and for SNPs with the highest 5% of F_{ST} values in the stringently filtered dataset. Minimum and maximum F_{ST} values are shown in parentheses.

Supplementary Table 2. **Measures of genetic diversity for mitochondrial (nucleotide diversity, π), Z intron (π), autosomal introns (observed heterozygosity H_o/expected heterozygosity H_e) and ddRAD SNPs (H_o/H_e).**

	mtDNA			Z intron			seven autosomal introns				SNPs			
	N_i	N_h	π	N_i	N_a	π	N_i	N_a	H_o	H_e	N_i	N_a	H_o	H_e
Chihuahuan	28	16	0.0116	20	4	0.0004	12-16	2-9	0.431	0.532	10-12	2	0.395	0.384
Common	421	81	0.0324	221	9	0.0006	95-98	4-15	0.444	0.515	42-47	2	0.232	0.247
California	54	15	0.0183	44	6	0.0001	14-15	2-7	0.376	0.441	5-6	2	0.319	0.338
Holarctic	161	39	0.0184	87	3	0.0001	40-42	3-11	0.409	0.472	17-21	2	0.241	0.257
west US	206	50	0.0338	90	7	0.0005	40-41	3-11	0.506	0.536	15-20	2	0.249	0.257

Number of individuals (N_i); Number of haplotypes (N_h); Number of alleles (N_a). Values for autosomal introns and SNPs represent ranges for N_i and N_a and means for H_o, and H_e calculated across the seven autosomal introns and 1205 SNPs.
