# Supplementary material for: Genomic evidence of speciation reversal in ravens
Source: Nat Commun. 2018 Mar 2;9:906. doi: 10.1038/s41467-018-03294-w (PMC5834606; doi:10.1038/s41467-018-03294-w)
Supplement: Supplementary file 2 — Description of Additional Supplementary Files [file 41467_2018_3294_MOESM2_ESM.pdf]

Description of Additional Supplementary Files

File Name: Supplementary Data 1
Description: Details of specimens examined. Acronyms are as follows: HOL, Holarctic; CAL, California; west, western USA; AMNH, American Museum of Natural History; ANSP, Academy of Natural Sciences, Philadelphia; BMNH, Bell Museum of Natural History; BRTC, Texas A&M Biodiversity Research and Teaching Collections; CM, Carnegie Museum of Natural History; CUMV, Cornell University Museum of Vertebrates; DMNH, Delaware Museum of Natural History; DMNS, Denver Museum of Nature and Science; FMNH, Florida Museum of Natural History; KU, Kansas University; LSUMNZ, Louisiana State University, Museum; MVZ, Museum of Vertebrate Zoology, University of California, Berkeley; NAU, Northern Arizona University; NCSM, North Carolina State Museum of Natural Sciences; NYSM, New York State Museum; OMNH, Sam Noble Oklahoma Museum of Natural History; RAM, Royal Alberta Museum; RSM, Royal Saskatchewan Museum; SDSU, San Diego State University; UA, University of Arizona; UAM, University of Alaska Museum; UBCBBM, University of British Columbia Beaty Biodiversity Museum-Cowan Tetrapod Collection; UCSB, University of California Santa Barbara; UMMZ, University of Michigan Museum of Zoology; UNMMSB, University of New Mexico, Museum of Southwestern Biology; USNM, Smithsonian National Museum of Natural History; UWBM, University of Washington Burke Museum; Y denotes samples sequenced in this study for mtDNA, Z intron (*ACO1*), autosomal introns (*RHOD, IRF2, PCBD, MYO2, RPL5, VIM, CLOCK*) and ddRADs; Y* denotes mtDNA sequences from previous studies acquired from GenBank; X denotes samples with failed ddRAD runs that were excluded from bioinformatic analyses. All sequences have been deposited on GenBank under the following accessions—mtDNA control region: MG594926 - MG595149; *ACO1*: MG591008 - MG591247; *RHOD*: MG583407 - MG583517; *IRF2*: MG590382 - MG590504; *PCBD*: MG590505 - MG590618; *MYO2/MB*: MG590619 - MG590732; *RPL5*: MG590733 - MG590880; *VIM*: MG590881 - MG591007; *CLOCK*: MG583286 - MG583406. Raw ddRAD reads were deposited in Sequence Read Archive (SRA) under BioSample accession numbers SAMN08118047-SAMN08118108.
